# Vinification without *Saccharomyces*: Interacting Osmotolerant and “Spoilage” Yeast Communities in Fermenting and Ageing Botrytised High-Sugar Wines (Tokaj Essence)

**DOI:** 10.3390/microorganisms9010019

**Published:** 2020-12-23

**Authors:** Hajnalka Csoma, Zoltán Kállai, Zsuzsa Antunovics, Kinga Czentye, Matthias Sipiczki

**Affiliations:** 1 Department of Genetics and Applied Microbiology, University of Debrecen, 4032 Debrecen, Hungary; hajnalka.csoma@outlook.com (H.C.); lepkekutato@gmail.com (Z.A.); czkinga12@gmail.com (K.C.); 2 Research Institute for Viticulture and Oenology, 3915 Tokaj, Hungary; kallai.zoltan@tarcalkutato.hu

**Keywords:** yeast, *Zygosaccharomyces*, *Candida*, wine, fermentation, osmotolerant, genetic diversity, antagonism, Tokaj

## Abstract

The conversion of grape juice to wine starts with complex yeast communities consisting of strains that have colonised the harvested grape and/or reside in the winery environment. As the conditions in the fermenting juice gradually become inhibitory for most species, they are rapidly overgrown by the more adaptable *Saccharomyces* strains, which then complete the fermentation. However, there are environmental factors that even *Saccharomyces* cannot cope with. We show that when the sugar content is extremely high, osmotolerant yeasts, usually considered as “spoilage yeasts“, ferment the must. The examination of the yeast biota of 22 botrytised Tokaj Essence wines of sugar concentrations ranging from 365 to 752 g∙L^−1^ identified the osmotolerant *Zygosaccharomyces rouxii*, *Candida (Starmerella) lactis-condensi* and *Candida zemplinina* (*Starmerella bacillaris*) as the dominating species. Ten additional species, mostly known as osmotolerant spoilage yeasts or biofilm-producing yeasts, were detected as minor components of the populations. The high phenotypical and molecular (karyotype, mtDNA restriction fragment length polymorphism (RFLP) and microsatellite-primed PCR (MSP-PCR)) diversity of the conspecific strains indicated that diverse clones of the species coexisted in the wines. Genetic segregation of certain clones and interactions (antagonism and crossfeeding) of the species also appeared to shape the fermenting yeast biota.

## 1. Introduction

The conversion of grape must to wine is the result of the joint activities of multiple yeast species. The spontaneous fermentation process starts with a mixed community of yeast species that are determined primarily by the yeast populations colonising the harvested grape and the winery environment (e.g., [1,2,3]). The composition of the community rapidly changes in the fermenting must because most non-*Saccharomyces* yeasts die and strains of the species *Saccharomyces cerevisiae*, *Saccharomyces uvarum* and genomic chimeras of various *Saccharomyces* species take the lead and drive the fermentation to completion. The success of the *Saccharomyces* strains is attributable to their overall better resistance and adaptability to the rapidly changing environmental factors (e.g., high levels of ethanol and organic acids, low pH values, scarce oxygen availability and depletion of certain nutrients) compared to non-*Saccharomyces* yeasts (reviewed in [1,4]). Only a few non-*Saccharomyces* strains can persist until the end of fermentation (e.g., [5,6,7,8,9,10]). 

The non-*Saccharomyces* species can display either beneficial or detrimental (“spoiling”) activities, but many of them have both. The latter play positive roles in certain phases of the fermentation process and negative roles in other phases or have a positive impact on the quality of certain types of wines and a negative effect on the quality and stability of other types of wines (for recent reviews, see [11,12,13,14,15]). Nevertheless, for practical reasons, the yeasts that can cause problems are usually considered spoilage microorganisms, even if they also have properties that can beneficially modify the sensory quality of the wine. The most frequently occurring yeasts with (at least occasional) wine spoilage effects belong to the species *Brettanomyces bruxellensis (Dekkera bruxellensis)* [16,17], *Zygosaccharomyces bailii* (*Saccharomyces bailiii*), *Z. rouxii* (*Saccharomyces rouxii*, *Saccharomyces osmophilus*), hybrids/chimeras of various *Zygosaccharomyces* species (e.g., [18,19,20,21,22]), *Candida lactis-condensi* (*Torula lactis-condensi*, *Torulopsis lactis-condensi*, *Starmerella lactis-condensi*) [23], *Candida zemplinina* (*Saccharomyces bacillaris*, *Torulopsis bacillaris*, *Starmerella bacillaris*) [10,23,24], *Hanseniaspora osmophila* (*Kloeckeraspora osmophila*, *Kloeckera corticis*) [25], *Pichia anomala* (*Candida pelliculosa*, *Hansenula anomala*) [26], *Pichia membranifaciens* (*Candida valida*) [27,28,29], *Rhodotorula mucilaginosa* [30], *Saccharomycodes ludwigii* [31,32], *Kregervanrija fluxuum* (*Pichia fluxorum*) [29] and *Candida apicola* [12]. Several of these species are osmotolerant or even osmophilic (e.g., [6,24,33,34,35]) and pose a threat to the stability of aging sweetened wines and wines containing higher levels of residual sugar, as well as to other high-sugar beverages, fruit juice concentrates, sugar confectionery products, honey, dried fruit and jams (e.g., [36,37,38,39,40]). 

Although non-*Saccharomyces* yeasts can occasionally become dominating, they are generally not capable of completing alcoholic fermentation on their own. In this study, we showed that osmotolerant yeasts that are considered unwanted spoiling organisms in wine-making can ferment high-sugar botrytised grape must without the contribution of *Saccharomyces*. Musts prepared from botrytised (nobly rotten) grapes usually have extremely high concentrations of sugars, which cannot be completely converted to ethanol and other metabolites during fermentation (for recent reviews, see [41,42]). Noble rotting requires specific microclimatic conditions that allow for the destruction of the skins and the internal structures of the ripening berries by the invading hyphae of *Botrytis cinerea* but prevents the fungus from causing malevolent sour rotting. Instead, the berries lose water through the *Botrytis*-generated lesions. Due to water evaporation, the sugar concentration in the juice of the shrivelling grapes increases drastically and the must prepared from these grapes will have a high sugar content [43]. More than 30 wine regions located in the cooler part of the wine-producing zones around the globe are known to have climatic conditions that allow for noble rot and the production of botrytised and/or *Botrytis*-affected wines [44]. The region with the longest documented history of botrytised wine production in Europe is Tokaj (Tokay) [45,46]. One of the most specific Tokaj wine brands that is unique in the world is Essence (Eszencia, Esencia), which is made from the juice that seeps out spontaneously from the harvested nobly rotten berries stored in large containers [47]. Its sugar content can exceed 700 g∙L^−1^, which is a concentration that is inhibitory to most yeasts occurring in various stages of the fermentation of other types of wines. Because of the extremely high sugar content, Essence fermentation lasts for years and produces little more than 5 to 7% alcohol [47,48]. Preliminary studies of our laboratory have detected strains of the osmotolerant “wine spoilage” species Z. *bailii, Z. rouxii, C. lactis-condensi* and *C. zemplinina* in samples of Tokaj Essence wines [49] but neither the composition of the yeast populations nor the properties of the fermenting strains were investigated. Since then, the latter species have been transferred to the genus *Starmerella* under the new names *S. lactis-condensi* and *S. bacillaris* [50,51] but the “previous names” were retained as valid synonyms and have remained widely used in the literature. Because of their widespread use and because the taxonomic description of the species *C. zemplinina* was based on Tokaj yeasts, we will use the old species names in this study. 

Here, we report on an investigation of the yeast biota of 28 Essence wines. The composition of wine yeast microflora can be investigated using culture-dependent and culture-independent methods (for a review, see [52]). Neither can explore the entire complexity of the active yeast communities. The former cannot detect yeasts that do not grow on laboratory media or are in the so-called VBNC (viable but not culturable) state. The latter does not distinguish between the DNA of living and dead yeasts and thus overestimates the diversity of the yeast community that is actively participating in fermentation [53]. To overcome this problem, certain studies applied reverse-transcription PCR, which amplifies copies of RNAs (e.g., [54,55,56]). This method is based on the assumption that only living cells have detectable RNAs. However, as RNA can be easily degraded by contaminating RNAse, the reproducibility of the results can be low. Because of these shortcomings of the culture-independent methods, we opted for a culture-dependent procedure. Another advantage of culture-dependent methods is that the isolated yeasts can be subjected to genetic and physiological investigation. The taxonomic analysis of high numbers of isolates identified 13 species, all of which are known as “wine spoilage yeasts”. Three of them, *Z. rouxii*, *C. lactis-condensi* and *C. zemplinina* dominated the majority of the wines, indicating that these yeasts run the fermentation. The rest of the species were present sporadically as minor subpopulations, including four film-forming yeasts. The populations of the dominating yeasts consisted of clones differing in phenotypic properties and molecular patterns and were prone to segregation. 

## 2. Materials and Methods 

### 2.1. Culture Media and Reference Yeast Strain

Two types of solid media were used for yeast isolation and the maintenance of the isolates: YEA2 (1% yeast extract, 2% glucose and 2% agar) and YEA35 (YEA supplemented with 35% glucose). The liquid media were YEL (YEA2 without agar) and YPGL (YEL supplemented with 1% peptone). SMA [1% glucose; 2% agar; 0.5% (NH_4_)_2_SO_4_; 0.01% KH_2_PO_4_, MgSO_4_ and vitamins] containing 1% carbon source or 0.5% nitrogen source to be tested instead of glucose or (NH_4_)_2_SO_4_ was used for the phenotypic clustering of isolates. H_2_S production was tested on BiGGY Agar (Bismuth Sulphite Glucose Glycine Yeast; Oxoid). Acid production was examined on Custer’s chalk medium (0.5% yeast extract, 5% glucose, 2% agar, 0.5% calcium carbonate, pH 5.0) [57]. The composition of the medium (synthetic must) used in the microplate experiments was: 0.5% KH_2_PO_4_, 0.04% MgSO_4_·7H_2_O, 0.1% yeast extract and 0.1% vitamin solution (according to [58]), pH 3.5 (adjusted with tartaric acid) supplemented with various amounts of glucose and fructose in a 1:1 proportion. *Z. rouxii* CBS 732^T^ was used as a reference strain in the investigation of the molecular diversity of the Z. *rouxii* isolates.

### 2.2. Sample Collection and Analysis

Twenty-eight Essence wines in various stages of fermentation and aging were sampled in February and March 2020 in wineries of the Tokaj region (Table 1). Nine sampled wines were in bottled lots prepared for commercial distribution. Nineteen samples were taken from batches that were fermenting and/or ageing in stainless steel containers, 50 L glass balloons, ceramic amphoras or oak barrels at 11–15 °C. The samples were kept in the laboratory at 12 °C. 

The pH and the alcohol, total sugar, titratable acid, SO_2_, acetic acid and extract contents were measured according to the methods laid down in the Commission Regulation (EEC) No. 2676/90 of the European Union [59]. When the sample volume was not enough for these analytical methods, a Bruker Alpha FTIR spectrometer (Bruker Optic GmbH, Ettlingen, Germany) was used and the results were processed with the Bruker OPUS software. The viable yeast cell number (number of CFUs: colony-forming units) was determined by diluting the samples and spreading aliquots on YEA2 plates. The number of colonies was counted after 6 days of incubation at room temperature.

### 2.3. Yeast Isolation and Phenotypic Categorisation 

To obtain individual yeast colonies, loopful volumes of the Essence samples were streaked on YEA2 and YEA35 plates. To minimise the selection bias, the media were not supplemented with agents inhibiting the growth of non-yeast microorganisms or differentiating the types of yeasts. After 7 days of incubation at 25 °C, 50 to 200 colonies were randomly isolated from the plates to obtain collections of pure isolates (single-cell clones) reflecting the population structures of the yeast biota of the wines. From samples containing low numbers of viable yeasts, 10–30 µL aliquots were spread on both types of plates, either directly or from pellets obtained by centrifugation of 100 mL samples. The isolates were maintained on YEA2 and YEA35 plates, stored at 5 °C and reinoculated onto fresh plates every second month. 

The isolates were grouped by examining their growth morphology and the results of physiological taxonomic tests. Growth morphology (colour, surface ornamentation, production of pigmented halo in the medium) was examined by streaking the isolates on YEA2 plates and incubating at room temperature. The ability of the isolates to assimilate eight compounds (saccharose, galactose, raffinose, mannitol, maltose, cellobiose, glycerol, acetic acid) as carbon sources and two compounds (lysine and potassium nitrate) as nitrogen sources and to grow at 37 °C and on vitamin-free medium SMA plates was tested by replica-plating, as described previously [42]. In both examinations, we encountered the problem that certain isolates were phenotypically unstable. From the cultures of these isolates, we re-isolated single-cell clones. The re-isolation did not stabilise the variable phenotypic traits, indicating that these isolates were prone to segregation.

### 2.4. Molecular Taxonomy

For taxonomic identification of the isolates, total genomic DNA was extracted from overnight cultures grown in YEL and used for the amplification of the D1/D2 domains of the 26S rRNA genes with the primers NL-1 and NL-4, as described previously [24]. The resultant amplicons were purified with a Gel/PCR DNA Fragments Extraction Kit (Geneaid Biotech Ltd.), sequenced with the same primers (Microsynth AG, Balgach, Switzerland) and the sequences were used to determine the taxonomic positions of the isolates in two steps. First, similar sequences were identified in the INDSC (International Nucleotide Sequence Database Collaboration) databases using the MEGABLAST-querying service of NCBI (https://blast.ncbi.nlm.nih.gov/Blast.cgi). In the second step, the sequences of the isolates were compared via pairwise Blast alignment (using the blast2seq algorithm available at NCBI) with the D1/D2 sequences of the type strains of the species whose strains were found to be most similar in the MEGABLAST search. Sectors of segregating colonies of certain isolates were also subjected to D1/D2 analysis to verify their taxonomic identity.

### 2.5. Electrophoretic Karyotyping and Microsatellite-Primed PCR (MSP-PCR) Fingerprinting 

Chromosomal DNA was prepared from the cells of overnight YEL cultures in agarose plugs, as described by Nguyen et al. [60] for *Saccharomyces*. The plugs were washed in TE (Tris/EDTA) and inserted into wells of 1.1% agarose (chromosomal grade, Bio-Rad, Hercules, California, USA) gel prepared in a 0.5× TBE (Tris/Borate/EDTA) buffer. The chromosome-sized DNA molecules were separated using pulse-field electrophoresis in 0.5× TBE with a CHEF-Mapper apparatus (Bio-Rad). The running parameters were as follows: ramping for 300 s over 48 h and ramping for 600 s over 48 h at 3 V/cm in a TBE 0.5× buffer at 14 °C. Gels were removed from the tray, stained in an ethidium bromide bath (0.5 mg/100 mL) and destained in sterile water. For MSP-PCR fingerprinting, genomic DNA was extracted from overnight YEL cultures. The PCR reaction was carried out with the microsatellite oligonucleotide primer (GAC)_5_, as described by Baleiras-Couto et al. [61]. *Z. rouxii* CBS 732^T^ was used as the reference strain in both procedures.

### 2.6. Extraction and Restriction Fragment Length Polymorphism (RFLP) Analysis of Mitochondrial DNA

Mitochondrial DNA was extracted from cells of two-day YEL cultures according to Nguyen et al. [60] for *Saccharomyces*. A total of 10 µL of mtDNA was digested with the restriction endonuclease *Hae*III in a final volume of 20 µL, and the fragments were separated via electrophoresis in 1.2% agarose and 0.5× TBE. The band size was determined using the PyElph1.4 software for gel image analysis [62]. The reference strain was *Z. rouxii* CBS 732^T^.

### 2.7. Cluster Analysis of Molecular Patterns

From the karyotype, MSP-PCR and mtDNA-RFLP pattern binary matrices (1: band is present, 0: band is absent) were constructed and used for the distance calculation with the Dice coefficient [63]. The distance matrices were then analysed with the average-linkage hierarchical clustering algorithm UPGMA (Unweighted Pair Group Method with Arithmetic mean)using the service available at http://genomes.urv.es/UPGMA [64]. Dendrograms were visualised with the FigTree programme (http://tree.bio.ed.ac.uk). 

### 2.8. Phenotypic Characterisation via Drop Tests 

For drop tests on agar plates, cell suspensions (OD 0.1) were prepared in sterile water from pellets (washed once with sterile water) of centrifuged cultures of the isolates grown in YPGL for one day at 25 °C. Aliquots (10 µL) of the suspensions were dropped on the test media, as described below. All tests were carried out in duplicate in two independent experiments.

#### 2.8.1. Determination of the MIC (Minimal Inhibitory Concentration) of Glucose, Ethanol and Potassium Bisulfite (K_2_S_2_O_5_) 

Samples of the suspensions were dropped on the surface of YEA plates supplemented with various concentrations of glucose (2, 30, 40, 50, 60 and 70%) and YEA2 plates containing various amounts of ethanol (0, 2, 3, 4, 6, 8, 10, 12 and 14%) or K_2_S_2_O_5_ (100, 200, 300, 400, 500 and 600 mg/L). The plates were incubated at 25 °C, and the growth of the isolates was monitored for ten days.

#### 2.8.2. Growth at Various Temperatures

To compare the effect of temperature on the growth of the isolates, samples of the suspensions were dropped on YEA2 plates, which were then incubated at 20, 25, 30 and 37 °C for ten days. 

#### 2.8.3. H_2_S Production

To examine the hydrogen sulphide production, samples of the suspensions were dropped on BiGGY Agar plates. The plates were incubated at 25 °C and the changes in the colony colour were monitored for 10 days. The intensity of the colour, reflecting the intensity of H_2_S production, was graded using the following visual colour scale: 1 (white), 2 (cream), 3 (light brown), 4 (brown), and 5 (dark brown). 

#### 2.8.4. Organic Acid Production

The intensity of the acid production was examined by culturing the isolates on Custer’s chalk plates. Samples of the suspensions of the isolates were dropped on the plates and incubated at 25 °C. On the tenth day, the width of the dissolution zones around the colonies was measured.

### 2.9. Examination of Biofilm Formation

Loopful amounts of cells of cultures grown on YEA2 plates were suspended in 30 mL of a test medium in a 50 mL Erlenmeyer flask. Two types of test media were used: YEL containing 2% glucose and YEL containing 50% glucose. The inoculated medium was incubated without agitation at room temperature for one week. If a pellicle was formed, the sample was taken from the surface of the medium for microscopic examination. Cells were viewed and photographed with an Olympus BX51 microscope and a DP70 digital camera.

### 2.10. Growth Assay with Microplates

Growth of the isolates in synthetic must supplemented with various amounts of sugar (fructose and glucose in 1:1 proportion) was examined in 96-well microplates. The sugar concentrations in the test media were 20, 50 and 60%. For inoculation, one-day-old precultures grown in the medium containing the same sugar concentrations were used. The precultures were centrifuged and the pellets were resuspended in the test media to obtain the initial optical density 0.1 (A_590_). The wells of the microplates were filled with the suspensions (200 µL in each well) in three replicates. The growth of the yeast strains in the wells at 25 °C was monitored with a SPECTROstar Nano Microplate Reader (BMG Labtech, Offenburg, Germany) by measuring the absorbance at A_590_ (no. of flashes per well and cycle were 22) at regular time intervals (10,000 s) for 5 days. Before each cycle, the plate was shaken at 300 rpm for 30 s.

### 2.11. Interaction and Growth Competition Tests

The isolates were tested for interactions on a solid medium, as described previously [42]. Dense suspensions were prepared in 2 mL of sterile water from 7- to 10-day-old cultures grown on YEA2. Then, YEA2 plates were individually flooded with the suspension of each isolate to produce homogeneous layers (lawns) of cells on the surface of the medium. The rest of the suspension was poured off. After drying the surface of the medium, loopful amounts of other isolates were smeared on the plates in spots of ≈1 cm in diameter. The plates were then incubated at 20 °C for three weeks and examined at regular time intervals for the growth intensity of the spots and the lawn around the spots. Poor spot growth indicated that the lawn had an antagonistic effect against the spot. Clear zones and zones of poor growth around the spot indicated that the spot had an antagonistic effect against the lawn. If the spot facilitated the growth of the lawn, the latter formed a thick ring of intense growth around the spot.

The relative growth rates (competition) of the isolates in the mixed populations were examined in YEL containing 2% glucose and in YEL supplemented with 35% glucose. In both media, pairs of isolates representing different species were examined. For each pair, a mixed culture containing 5 × 10^5^ cells∙mL^−1^ of both strains and two pure control cultures of the strains containing 10^6^ cells∙mL^−1^ were set up in 30 mL YEL. The cultures were incubated on a gyratory shaker at 20 °C. After 48 h, the density of the cultures was determined via cell counting in a Bürker chamber and diluted aliquots were spread on plates of two types of selective SMA media from each culture. The media differed in composition such that each strain could form colonies on only one of them. For example, medium A contained trehalose and medium B contained mannitol as a carbon source in the competition test of *Z. rouxii* and *P. membranifaciens*. The former formed colonies on medium A, the latter formed colonies on medium B because *Z. rouxii* is mannitol^+^ and trehalose^−^, whereas *P. membranifaciens* is mannitol^−^ and trehalose^+^. Thus, the cells of each control culture could produce colonies on only one medium, whereas the cells of the mixed culture formed colonies on both. The proportion of the colony numbers produced by samples of the mixed culture spread on medium A and medium B showed whether the strains grew equally well when mixed (proportion ≈ 1:1) or differed in growth rate (proportion ≠ 1:1). When a *Metschnikowia* isolate was involved in the test, one of the media was YEA2-supplemented with 0.02 mg/mL FeCl_3_, on which its cells produced maroon colonies. When the isolates differed in thermotolerance, one of the selective conditions was incubation at 35 °C.

## 3. Results

### 3.1. Sampling and Sample Characterisation

Samples were taken from wines covering a timespan of 35 years (Table 1). Six of the eight wines produced in the 2019 vintage had high CFU counts and also showed other signs (e.g., bubbles) of active fermentation. The fermentation of the Tokaj Essence is a slow process lasting for months or even years without a clear definition of completion. The rest of the 2019 vintage wines either had no culturable yeasts (wine 13) or the count of the CFUs was extremely low (wine 15). The low yeast activity in these wines could be due to excessive sulphurisation to protect the wine against the oxidation that killed the natural microflora in the early stage of fermentation (both wines had 0% alcohol). No viable yeasts were detected in four bottled wines. The sugar content ranged from 365 to 752 g∙L^−1^ and the alcohol level of the wines containing culturable yeasts varied between 0.39 and 5.53%. Low alcohol content is another characteristic feature of Essence; it rarely exceeds 7% [47]. The pH did not vary significantly despite the considerable diversity in acid content.

### 3.2. Isolation and Phenotypic Categorisation of Yeasts

From the 22 wines, yeasts could be isolated as individual colonies formed on media containing either 2% (on YEA2) or 35% (on YEA35) glucose. The latter concentration corresponded to the lowest sugar content in the examined wines. We used two sugar concentrations for yeast isolation because certain osmotolerant yeasts grow better in high sugar content (e.g., [65]). Altogether, 3209 colonies were isolated. The isolates were then divided into groups (clusters) on the basis of 10 taxonomically relevant physiological properties and the morphology of their cultures on YEA2 plates. 

### 3.3. Taxonomic Identification

The taxonomic affiliations of the selected representatives of all clusters of isolates in each wine were determined using the standard D1/D2-domain-based method [66]. We opted for this method because the potential alternatives, such as ITS sequencing and MALDI-TOF, are less efficient and/or less specific. Fewer ITS sequences than D1/D2 sequences are available for yeasts in databases, and the number of available MALDI-TOF reference spectra is still limited [67]. Except for five isolates, all showed 100% D1/D2 sequence identity with the type strains of known species (Table S1). The exceptions were isolate 8-1, which differed from the type strain of *C. lactis-condensi* by one substitution, and the isolates that produced maroon-red colonies. The latter had a few ambiguous nucleotides in their D1/D2 sequences, which prevented the determination of their exact taxonomic position. Nevertheless, all showed high similarity to the D1/D2 sequences of the pulcherrimin-producing *Metschnikowia* yeasts referred to as the *pulcherrima* clade [68]. The phenotypical clusters could be assigned to 13 species by sequencing the D1/D2 domains of their 99 representatives (Table S1). Interestingly, certain clusters turned out to have identical D1/D2 sequences. This finding indicated that phenotypically different conspecific clones were present in the yeast biota of certain wines (see Section 3.5). 

**Figure 1:**
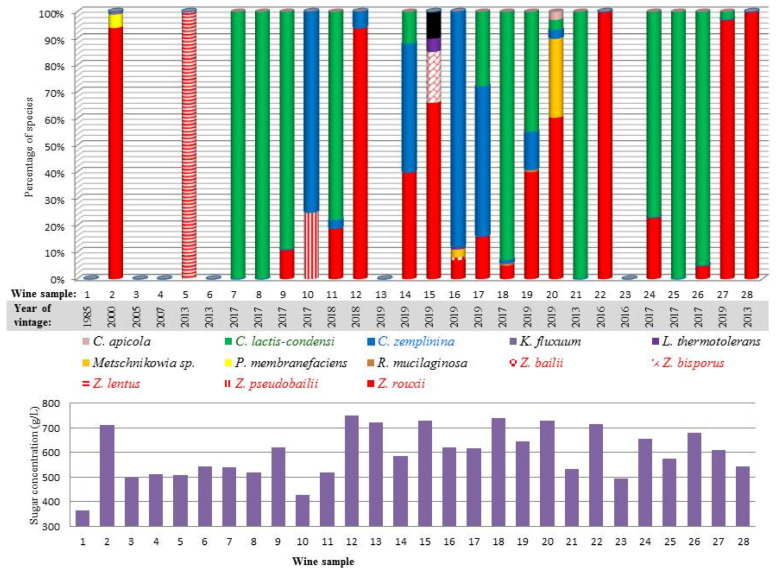
Composition of the yeast biota and the sugar content in the wine samples.

The relative abundance of the species in the individual wines is shown in Figure 1. The most abundant yeasts were *C. lactis-condensi* and *Z. rouxii*. In four wines (7, 8, 21 and 25), all isolates belonged to *C. lactis-condensi*, and from two wines (22 and 28), only *Z. rouxii* was isolated. *C. zemplinina* was detected in nine wines, where in two of them (10 and 16), it was detected as the dominating but not exclusive species. The genus *Zygosaccharomyces* was represented by four more species (*Z. bailii*, *Z. bisporus*, *Z. lentus* and *Z. pseudobailii*) but each of them was found in one wine only. Their proportion in the population varied between 1 and 25%, with the exception of *Z. lentus*, which formed 100% of sample 5. The rest of the species (*H. osmophila*, *K. fluxuum*, *Lachancea thermotolerans*, *Metschnikowia pulcherrima* clade sp., *P. membranifaciens*, *C. apicola*, *R. mucilaginosa*) occurred only sporadically. The same species in roughly identical proportions were identified on both types of media. Surprisingly, no *Saccharomyces* were found among the 3209 isolates. 

### 3.4. Diverse Osmotolerance of the Species

As shown above, all but one wine were dominated by *Z. rouxii*, *C. lactis-condensi* or *C. zemplinina*. The exception was the bottled wine 5, from which only *Z. lentus* colonies were isolated. It is reasonable to assume that their dominance is attributable to properties that render them able to cope with the harsh environment in Essence and give them a selective advantage over (the undetected) *Saccharomyces* and the other yeasts found in the samples. As the most noticeable difference between the Essence juice and the normal grape juice is the very high sugar content in the Essence juice, we compared the osmotolerance of isolates representing the species. Except for the *K. fluxuum*, *P. membranifaciens* and three *C. lactis-condensi* isolates, all grew on the agar medium supplemented with 70% glucose but the growth of the *Metschnikowia* strains was very poor (Table 2).

**Figure 2:**
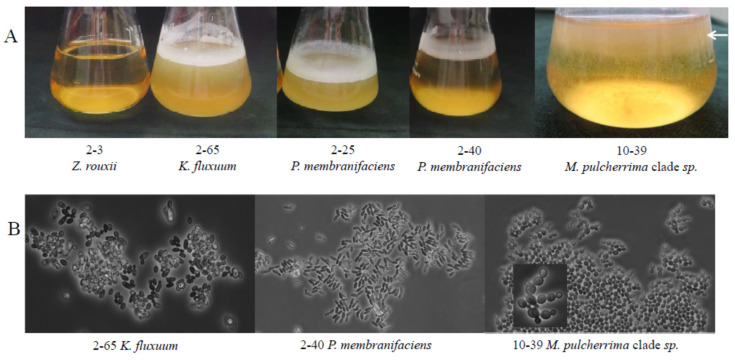
Surface film (pellicle) formation: (**A**) films on a liquid medium and (**B**) microscopic morphology of cells in samples taken from films. The white arrow shows descending cells/pseudohyphae after gentle shaking of the *Metschnikowia* culture.

As various yeast species with spoilage potential can form biofilms (e.g., [69,70]), we tested the isolates assigned to these species and representatives of the other species for biofilm formation by culturing them in liquid media without agitation. All *K. fluxuum*, *P. membranifaciens*, *R. mucilaginosa* and *Metschnikowia* isolates, but none of the isolates of the other species, formed pellicles (films) on the surface of the medium at both glucose concentrations (2 and 50%) (Figure 2). The *K. fluxuum* and *P. membranifaciens* films consisted of firmly aggregated cells, whereas the *Metschnikowia* and *R. mucilaginosa* films were formed by loosely connected short pseudohyphae. Even gentle shaking disrupted the *Metschnikowia* and *R. mucilaginosa* films and caused a massive sinking of cells.

**Figure 3:**
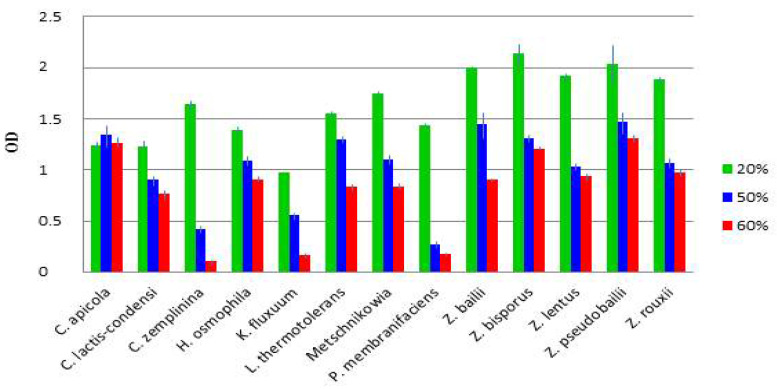
Microplate growth assay. The columns show the average optical density (OD) values calculated from the results shown in Table S2.

The effect of the sugar concentration on the growth of selected representatives of the species was also tested in artificial must. The optical density of the microplate cultures after five days (119 h 27 min) of incubation is shown in Table S2 and Figure S1. Figure 3 compares the growth of the species. All but one strain grew much better at 20% sugar than at higher sugar concentrations. The exception was *C. apicola*, which seemed to be slightly osmophilic. Remarkably, in these tests, the *C. zemplinina* isolates proved highly sensitive to 50 and 60% sugar, and the *Zygosaccharomyces* isolates grew much better than the strains of the other species at 20% sugar.

### 3.5. Intraspecies Phenotypic Diversity

**Figure 4:**
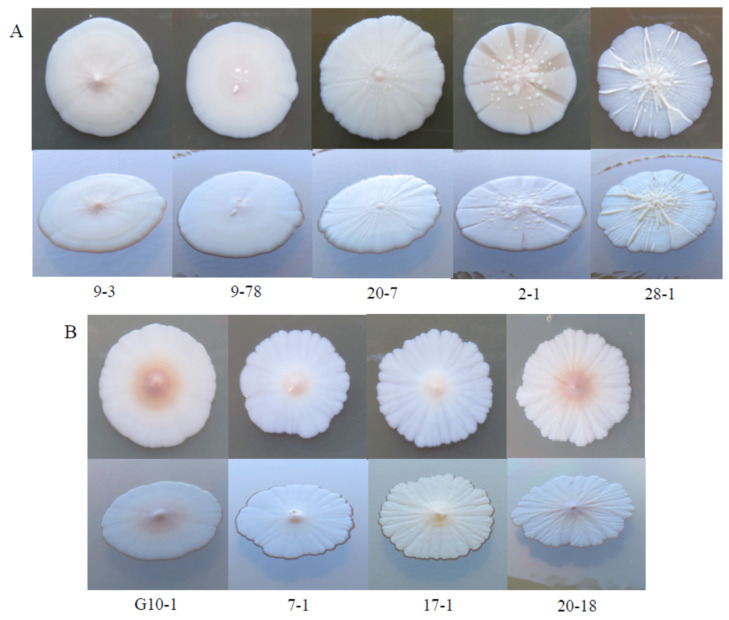
Diverse colony morphologies: (**A**) *Z. rouxii* and (**B**) *C. zemplinina*.

As mentioned above, certain phenotypically different groups of isolates proved to be conspecific in the molecular taxonomic examinations. One of the variable traits was the morphology of the colonies. Figure 4 shows how diverse colony morphologies were observed among the *Z. rouxii* and *C. zemplinina* isolates.

The *Z. rouxii* isolates were also variable in their utilisation of maltose as a carbon source and the ability to grow at 37 °C (Table 3). Further differences were detected in their growth intensity on media containing galactose, glycerol or mannitol as carbon sources or lysine as the nitrogen source. To further explore the diversity of the *Z. rouxii* isolates, we compared the ability of 46 isolates to grow at elevated concentrations of glucose, ethanol and K_2_S_2_O_5_. All grew at 70% glucose but the MIC (minimal inhibitory concentration) of ethanol and K_2_S_2_O_5_ varied considerably (10–14% and 300–800 mg∙L^−1^, respectively). The isolates also proved diverse in their acid secretion and H_2_S production.

**Figure 5:**
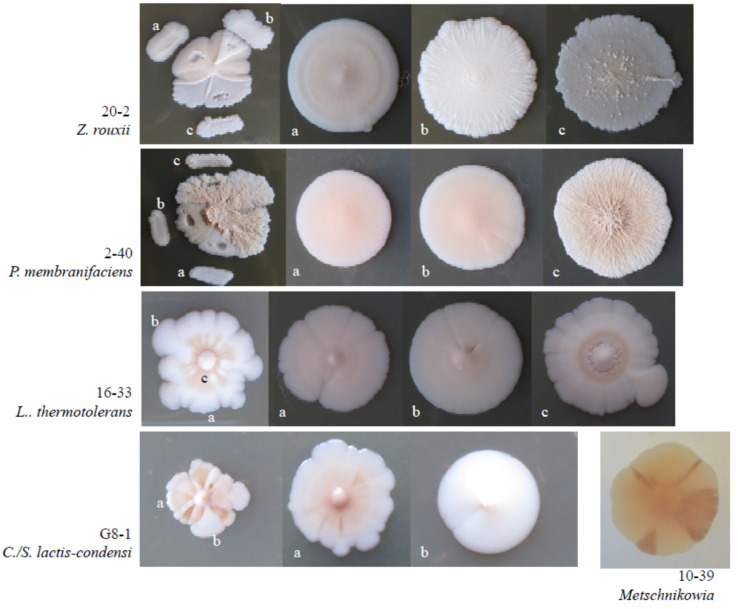
Segregating colonies: (**a**–**c**) sectors and the colonies formed by cells 324 of the sectors transferred on fresh agar plates.

Apart from their morphological diversity (Figure 5), the colonies of certain isolates segregated into sectors which were identical in D1/D2 sequences. Upon inoculation on fresh medium, the sectors usually retained their morphology but were prone to recurrent segregation. This phenomenon was observed among the *Z. rouxii*, *L. thermotolerans*, *C. lactis-condensi, P. membranifaciens* and *Metschnikowia* isolates. 

### 3.6. Intraspecies Molecular Diversity in Z. rouxii 

**Figure 6:**
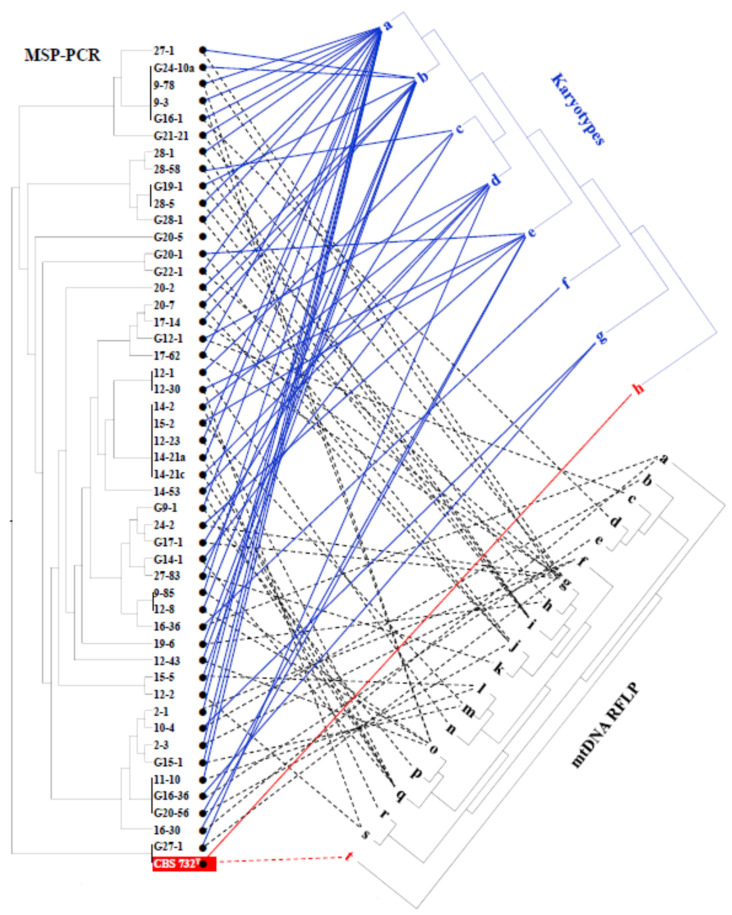
Molecular diversity of the *Z. rouxii* isolates. Comparison of the positions of the isolates on microsatellite-primed (MSP)-PCR, karyotype, and mtDNA restriction fragment length polymorphism (RFLP) dendrograms.

Next, we asked whether differences can also be found in the genomes of the isolates. Towards this end, we compared the karyotypes, the MSP-PCR patterns of the nuclear genomes and the RFLP patterns of the mitochondrial genomes of the *Z. rouxii* isolates. The isolates proved highly diverse in all tests. The highest diversity was detected with MSP-PCR (Figure 6). The total number of MSP-PCR patterns was 26, of which, 19 were unique. Seven patterns were shared by two to five isolates. The number of the mtDNA patterns was 19 (Figure S2) and 7 karyotypes (Figure S3) could be distinguished. A comparison of the dendrograms derived from the banding patterns revealed low similarity in their topology (Figure 6). Isolates showing identical MSP-PCR patterns were grouped in different clusters on the karyotype and mtDNA dendrograms. For example, the members of the largest group of identical MSP-PCR patterns had three different karyotypes and two different mtDNA patterns. The type strain of *Z. rouxii* (CBS 732^T^) shared its MSP-PCR pattern with one of the isolates but had a unique mtDNA pattern and a unique karyotype. Strains isolated from the same wine rarely had identical MSP-PCR and mtDNA RFLP patterns, indicating that different clones constituted the *Z. rouxii* population. For example, the 8 strains isolated from wine 12 had 5 karyotypes, 5 MSP-PCR and 5 mtDNA-RFLP patterns, and the pattern identity from one test did not always correlate with the identity from the other tests.

### 3.7. Interactions and Competitions of Isolates

**Figure 7:**
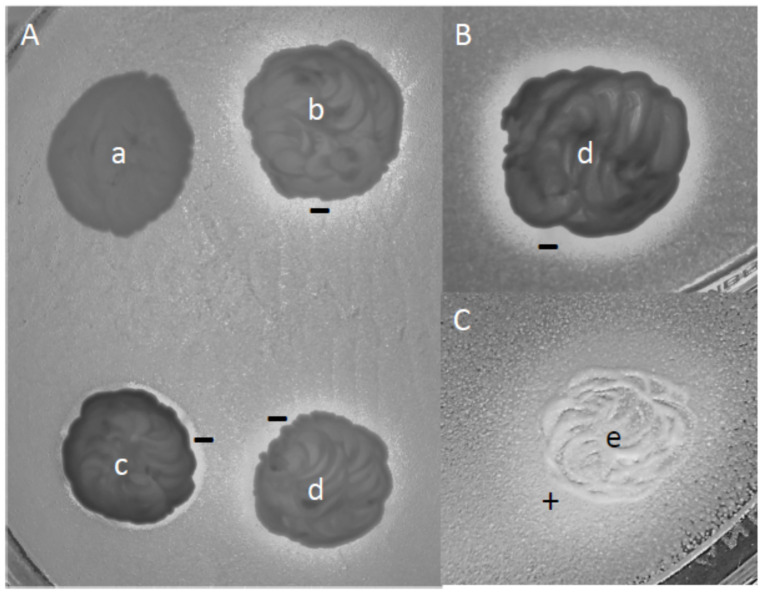
Interactions of isolates on an agar medium. Lawn: 20-2 *Z. rouxii* (**A**), G8-1 *C. lactis-condensi* (**B**) and 16-33 *L. thermotolerans* (**C**). Colonies: 15-1 *H. osmophila* (a), 16-33 *L. thermotolerans* (b), 20-1 *Metschnikowia* sp. (c), 15-11 *L. thermotolerans* (d) and *Z. pseudobailii* (e). −: reduced growth (antagonism). +: increased growth (crossfeeding).

In mixed populations, microorganisms can interact and their interactions then shape the population kinetics. To find out whether the yeasts of the Essence wines can interact in any way, we tested representative isolates to see whether they affected each other’s growth on agar plates (Table 4, Figure 7). The *Lachancea* colonies inoculated on tester lawns reduced the growth of the lawns (turbid inhibition zones) of almost all other isolates. The exception was one of the *P. membranifaciens* strains, whose lawn was not affected by one of the *L. thermotolerans* isolates. Interestingly, when the *Lachancea* isolates were used as lawns, they formed halos of thicker growth around the *Pichia* and *Zygosaccharomyces* colonies, presumably due to cross-feeding by nutrients or growth factors released by the colonies. Clear inhibition zones (the lawn did not grow within the zone) were rarely observed and were narrow. Remarkably, the *Z. rouxii* isolates reduced the growth of the lawns of the *H. osmophila*, *K. fluxuum* and *P. membranifaciens* isolates, as well as one of the *C. lactis-condensi* isolates. When pairs of isolates were cultured in liquid media, their proportion changed during the 48 h long test period (Table 5). Only combinations were examined in which the cells of isolates could be enumerated by different colony-forming abilities in different conditions (on media supplemented with different nutrients and by incubation at different temperatures). At 2% sugar, almost all tested isolates grew faster than *Z. rouxii*. Five non-*Zygosaccharomyces* isolates drastically overgrew the *Z. rouxii* isolate. At 30% sugar, the proportions changed in favour of *Z. rouxii* in certain combinations. The largest changes were detected in the mixed cultures of *Z. rouxii* with *C. zemplinina and C. lactis-condensi*: at 2% glucose, the mixed cultures contained more *Candida* cells, while at 30% glucose, the number of *Z. rouxii* cells was higher. Supplementation of the medium with 30% glucose caused 18-fold (compared to *C. lactis-condensi*) and 60-fold (compared to *C. zemplinina*) increases in the relative abundance of the *Z. rouxii* cells.

## 4. Discussion

Yeast isolation was attempted from samples taken from 28 Tokaj Essence wines ranging in age from half a year to 35 years and in sugar content from 365 to 752 g∙L^−1^. Viable yeasts were found in 22 wines, but the number of colony-forming units showed a high diversity. The wines made from the juice of nobly rotten grapes harvested during last year’s (2019) vintage had high numbers of culturable yeast cells, indicating that they were in the phase of active fermentation despite the fact that 5 to 6 months had passed since the harvest. The only exception was a wine that was heavily treated with sulphur dioxide against oxidation. The older wines were heterogeneous in viable yeast content. As the fermentation of the Tokaj Essence is a slow process lasting for months or even years without a clear definition of completion (alcohol content, residual sugar), it is unclear whether the older wines having higher numbers of CFUs were still in the primary fermentation stage or in secondary (re)fermentation. All but one of the bottled wines contained no viable yeast cells. From the 22 wines, 3209 yeast colonies were isolated via a random selection. To avoid immense sequencing, the isolates were first divided into phenotypic categories and then only representatives of the categories were sequenced. This strategy previously proved efficient in investigating yeast communities of botrytised grapes [42]. The analysis of the D1/D2 sequences amplified from 99 isolates identified 13 species that are all known to cause microbial spoilage in high-sugar beverages and food products (e.g., [6,33,71,72,73,74]) and occur in fermenting and aging wines of high sugar contents (e.g., [6,10,75,76,77]). 

### 4.1. Dominant and Associated Yeasts

The yeast populations of the wines were dominated by two groups of related yeasts. *Zygosaccharomyces* species were found in 18 out of the 22 wines containing culturable yeasts, and strains of the closely related *C. lactis-condensi* and *C. zemplinina* were detected in 17 wines. The genus *Zygosaccharomyces* consists of fifteen species (NCBI Taxonomy, Oct, 2020), of which *Z. bailii, Z. bisporus, Z. lentus, Z. pseudobailii* and *Z. rouxii* were represented among the isolates. *C. lactis-condensi* and *C. zemplinina* are almost indistinguishable by conventional taxonomic tests and were recently transferred to *Starmerella*, a rapidly expanding genus that harbours yeast species that occur frequently on flowers and flower-visiting insects [51]. 

*Z. rouxii* was found in 16 Essence wines, in two as the only species and in five as the dominating yeast. The other *Zygosaccharomyces* species were much less abundant; each was only found in one wine, with each found in a different wine. From these results, it can be concluded that although five *Zygosaccharomyces* species were detected in the Essence wines, only *Z. rouxii* appeared to play a significant role in the vinification process. Its presence and high abundance in the majority of the examined wines is somewhat surprising because it is a rare component of the grape microflora in the Tokaj region [72], and its relative, *Z. bailii* is present much more frequently in high-sugar wines. The latter was previously detected in aging Sauternes wines [75], vino cotto [6] and in a sweet Tokaj wine [29], but in this study, it was only found as a minor component of the yeast population in a fermenting Essence from last year’s vintage. This yeast is thought to get into the wine from the grapes, particularly from berries damaged by rotting [9]. This can also be the case in the Essence wine because a previous study detected this species in botrytised berries in the Tokaj region [42]. *Z. bailii* is one of the most common spoilage microorganisms of high-sugar food and wine (for a recent review, see [73]), but it has also been reported to be beneficial for vinification [78,79,80,81,82]. Thus, its presence in Essence wine is not necessarily negative. The closely related *Z. pseudobailii* (differing from *Z. bailii* by only one substitution in the D1/D2 sequence) was found in a bottled wine together with three non-*Zygosaccharomyces* species. The examined bottle had a very low number of CFUs and did not show any sign of fermentation. This species has a chimeric (admixed) genome that is assumed to have evolved from the hybridisation of *Z. bailii* and an unidentified species of the genus [22]. *Z. bisporus* was also found in an Essence wine from last year’s vintage but that wine had a very low number of culturable yeasts and did not show signs of fermentation. According to the information received from the winery, it was heavily sulphurised in order to prevent oxidation. The low yeast activity could be attributed to this treatment. *Z. bisporus* is closely related to *Z. bailii* and *Z. pseudobailii* [83,84]. It was described as spoilage yeast with intermediate features between *Z. rouxii* and *Z. bailii* [71,72] and has been isolated from both healthy grapes [54] and rotten grapes [85]. From one of the bottled Essence wines, only *Z. lentus* was isolated. This recently described species is characterised by a stress tolerance similar to that of *Z. bailii* but it grows slowly at low temperatures [86]. This property might account for its low abundance in the Essence wines, which are fermented and stored at low temperatures. The sporadic occurrence of the non-*rouxii Zygosaccharomyces* species in the Essence wines and their low abundance indicate that they only play minor roles (if any) in fermentation, but may affect the development of the sensorial properties of the wine during aging. 

*C. lactis-condensi* was the exclusive or the dominating species in 10 wines. In terms of the number of dominated wines, *C./S. lactis-condensi* seems to be the best-fitted yeast for propagation in and fermentation of the Essence wines. However, apart from being sporadically detected in spontaneous wine fermentation [23,87], little is known about its oenological properties. *C. zemplinina* constituted the majority in four wines. Both *C. lactis-condensi* and *C. zemplinina* were previously detected in sweet Tokaj wines, including Essence-type wines [23,24,49,88,89,90]. The latter has been found in many wine-growing regions of the world usually in botrytised [10,44,90] and sweet wines (e.g., [77,91,92,93]) and turned out to have a beneficial effect on wine quality in fermentations inoculated with mixed starters (for a review, see [94]). Because of these properties, it can be assumed to play a positive role in the fermentation of the Essence wines. Its close relative, *C apicola* (recently renamed to *S. apicola* [51]), was found as a minor component of the yeast biota in the wine populated by the most diverse yeast community (five species). This species has also been found in high-sugar substrates, including wines (e.g., [6,33,74]).

Low percentages of the yeasts isolated from two wines in the stage of active fermentation (last year’s vintage, high density of CFUs) showed taxonomic affiliation with the *pulcherrima* clade of *Metschnikowia,* but could not be assigned to any species because their D1/D2 sequences contained ambiguous nucleotides at certain positions. Di- and polymorphic nucleotides often occur in the D1/D2 sequences of pulcherrimin-producing *Metschnikowia* isolates when amplified directly from genomic DNA [68]. This phenomenon is attributable to inefficient homogenisation of the rDNA repeats. The analysis of the genome sequences of certain strains of the *pulcherrima* clade revealed that their rDNA is fragmented and thus evolves via the birth-and-death mechanism rather than via homogenisation, which is unusual in yeasts [68]. Yeasts belonging to this clade are ubiquitous on/in fruits, beverages and fruit-based food products (e.g., [85,95]) and were also found in botrytised grapes from the Tokaj region [42,96]. As the fermentation power of *Metschnikowia* is lower than that observed for other non-*Saccharomyces* species [15], the strains found in the Essence wines are not likely to significantly contribute to the fermentation process but they may modulate the composition of the wine. Strains of the *pulcherrima* clade have been found to affect wine quality, both positively and negatively (for recent reviews, see [15,85]).

The rest of the species were only found in very low numbers. A few colonies formed on the plates inoculated with samples of a 10-year-old wine dominated by *Z. rouxii* proved to be conspecific with the type strains of *P. membranifaciens* and *K. fluxuum. H. osmophila* and *L. thermotolerans* were detected as minor components of the yeast biota in a non-fermenting wine. The latter was also a minor associate (5%) of two *Zygosaccharomyces* species in a young fermenting wine. Very few *R. mucilaginosa* colonies (1–2%) were also found in a 3-year-old aging wine and a last-vintage wine. None of these yeasts were abundant enough to contribute significantly to vinification.

The abundances of *C. lactis-condensi* and *C. zemplinina* were comparable to that of *Z. rouxii*, which indicates that these three species were the major yeasts that drove the vinification process of the Essence wines.

### 4.2. Why These Yeasts Populate Essence Wines

The high sugar content of the Essence wines is a very harsh environment that is inhibitory for most yeasts normally participating in grape wine fermentation. The *Zygosaccharomyces* and *Candida* species identified in the Essence samples can cope with this condition because they tolerate high osmotic pressures (low water activity). In a recent comparative examination of over 600 hundred strains, *Z. rouxii* proved to be the most osmotolerant yeast among 151 species [35]. Its strains were able to grow at glucose concentrations as high as 5.5 M. The other *Zygosaccharomyces* species whose strains were detected in the Essence wines proved to be much less osmotolerant in those tests: the minimal inhibitory concentrations determined for *Z. bailii, Z. bisporus* and *Z. lentus* were 3.5–4.25 M, 3.25–4.5 M and 3.5–4 M, respectively. In light of these data, the higher propagation efficiency of *Z. rouxii* compared to the other *Zygosaccharomyces* species can be attributed to its higher osmotolerance. *Z. bailii* and *Z. bisporus* could only form minor components of the yeast populations because the sugar concentrations (≈3.4 M and ≈4.05 M) of their wines were only slightly lower than their MIC in the tests of Stratford et al. [35]. The wine from which only *Z. lentus* was isolated had a much lower sugar concentration (≈2.8 M) than the MIC (3.8 M) determined for this species in the aforementioned screening. Somewhat contradictorily with this data, in our tests, all *Zygosaccharomyces* isolates grew on the agar medium supplemented with 70% glucose (an MIC higher than 3.9 M) and in the artificial must containing 30% glucose and 30% fructose (≈3.3 M).

*C. apicola* was found to be somewhat more osmotolerant (MIC: 4.5 M) than the non-*rouxii Zygosaccharomyces* species by Stratford et al. [35] and more tolerant than *Z. rouxii* in our microplate assays, yet it was only found as a minor subpopulation in a wine dominated by *Z. rouxii*. The minimal inhibitory concentrations determined by Stratford et al. [35] for *Metschnikowia* sp. (2.75–3 M), *P membranifaciens* (2.75–2.9 M), *K. fluxuum* (2.45 M) and *R. mucilaginosa* (2.5–3.5 M) were much lower than the sugar concentrations of the Essence wines (except for the wine dominated by *Z. lentus*). *L. thermotolerans*, *C. lactis-condensi* and *C. zemplinina* were not examined in that study, but in other works, *C. zemplinina* proved more osmotolerant than *Z. bailii* [6] and *C. lactis-condensi* was as tolerant as *C. apicola* [34]. Little is known about the osmotolerance of *L. thermotolerans*. A recent study reported on more and less osmotolerant *L. thermotolerans* strains, with the former growing faster than the reference *S. cerevisiae* strain in a must containing 48% sugar (≈2.7 M) [97]. Consistent with the literature data on the different osmotolerance of *Z. rouxii*, *C. lactis-condensi* and *C. zemplinina*, the average sugar content of the Essence wines dominated by them was 681 g∙L^−1^ (≈3.8 M), 588 g∙L^−1^ (≈3.2 M), and 554 g∙L^−1^ (≈3.1 M), respectively. Thus, each of the three major yeasts of the Essence wines seems to have a range of sugar concentrations in which it can be more successful than the other species. However, this tendency was not corroborated by the microplate growth assays in which the isolates belonging to these *Candida* species attained a lower culture density than the *Z. rouxii* isolates both at 500 and 600 g∙L^−1^ sugar concentrations during five days of incubation. Nevertheless, none of the concentrations seemed to be restrictive to any of them because, in 12 of the wines, two of them or even all three were present simultaneously.

Because of the low osmotolerance of the species *K. fluxuum, Metschnikowia sp., P. membranifaciens*, and *R. mucilaginosa* [35], their strains probably could not propagate in the wines from which they were isolated. *R. mucilaginosa* could not grow in the wine either because it is an aerobic basidiomycetous yeast that is unable to gain energy by fermentation. Their presence can be attributed to their ability to form surface pellicles (biofilms). In the cool cellars, the humidity of the air condenses on surfaces and makes them wet. Condensation on the wine surface creates a thin layer of moisture that locally dilutes the wine (reduces its sugar concentration). Within the layer, the cells of these less osmotolerant species can propagate and form biofilms. Many yeast species can form biofilms by aggregating their cells and/or producing pseudohyphae [70]. We found that the *K. fluxuum* and *P. membranifaciens* biofilms consisted of adhered cells, and the *Metschnikowia sp.* and *R. mucilaginosa* isolates also formed pseudohyphae. The best-known examples of biofilm-forming yeasts in wine-making are the so-called “flor strains” of *S. cerevisiae* that rise to the surface of Sherry wine, where their cells switch from fermentative to oxidative metabolism and oxidise ethanol to precursors of molecules that are responsible for the specific sensorial properties of aging Sherry wines [98]. In a previous study, we found two *S. cerevisiae* “flor yeast” races (*capensis* and *aceti*) in aging Tokaj wines [99]. However, the function of the films of the non-*Saccharomyces* Essence yeasts may not be gaining energy from ethanol (an unlimited amount of sugar is available) but instead colonising the thin non-toxic environmental niche. Given their low abundance in the yeast biota, they are unlikely to affect the fermentation but may modulate certain sensorial properties of the wine. All low-abundance yeast species found in this study are known to have favourable abilities that can be exploited to improve the wine quality when inoculated in the wine as components of mixed starters (for recent reviews, see, e.g., [11,14,85,100]).

Except for the bottled wine having a low viable yeast number, from which only *Z. lentus* was isolated, the wines were dominated by one or the other of the species *Z. rouxii*, *C. lactis-condensi* and *C. zemplinina*, but in 12 wines, at least one of these three species was also present as a minor subpopulation. In two of the wines, none of them exceeded 50%. These results indicate that these species competed for dominance. How can one yeast prevail over the others when the conditions are not lethal to any of them (see Section 4.1)? Osmotolerance is undoubtedly an important factor in the competition, but it is by no means the only one. Although *C. lactis-condensi* is less osmotolerant than *Z. rouxii*, it dominated (by 93 %) a wine whose sugar concentration was much higher than the average sugar content of the wines dominated by *Z. rouxii*. On the other hand, only *Z. rouxii* colonies were isolated from a wine containing less sugar than the average sugar contents of the wines dominated by the *Candida* species. Thus, the actual proportions of the species can be the outcome of the interplay of several factors.

Such factors can be the interspecies interactions in the biota. Yeast strains can interact in many ways, such as by direct physical contact, competition for nutrients and the production and release of inhibitory compounds and toxins (for a review, see [101]). As for the species found in the Essence wines, *Z. bailii* [102,103] and *P. membranifaciens* (reviewed in [104]) were previously found to have strains producing killer toxins. Zygocin, the killer factor of a *Z. bailii* strain, was reported to strongly inhibit the growth of a *Z. rouxii* strain [103]. Alonso et al. [105] found that certain *P. membranifaciens* strains isolated from olive brines inhibited the growth of many (but not all) *Z. rouxii*, *Z. bailii* and *Z. bisporus* strains, but the sensitivity of the strains was diverse. The pulcherrimin-producing strains of the *pulcherrima* clade of *Metschnikowia* are known to inhibit the growth of other yeasts by immobilising the ferric ions in the environment [96]. A recent study also revealed antagonistic interactions between certain yeasts colonising Tokaj grapes. *Metschnikowia sp.*, *P. membranifaciens* and *Z. bailii* strains inhibited the growth of *L. thermotolerans; L. thermotolerans* strains and *Metschnikowia sp.* isolates had antagonistic effect (growth inhibition) against *Pichia membranifaciens* [42]. Like the grape yeasts, the Essence isolates also affected each other’s growth when tested on agar plates. Two types of interactions were observed: growth inhibition (reduced growth of the lawn around the colony of the other isolate) and crossfeeding (facilitated growth of the lawn around the colony of the other isolate). *L. thermotolerans* reduced the growth of almost all other isolates involved in the tests, but their own growth was facilitated around the colonies of most other isolates. These interactions can be attributed to competition for nutrients since the two *L. thermotolerans* isolates also inhibited each other (for inoculation of the colonies on the lawns, a large amount of cells were used, which could deplete the medium of certain nutrients around them very fast). The facilitated growth in the lawn of these isolates around the colonies of other isolates might be due to secreted nutrients and/or growth factors. As for the three major species, only one of the tested *C. lactis-condensi* strains showed reduced lawn growth around the *C. zemplinina* and *Zygosaccharomyces* colonies. Similar interactions could also operate in the Essence wine despite the very different environment in the wine.

One important difference is that the wine is a fluid environment in which the compounds can diffuse much easier. Therefore, we also tested the isolates for growth in mixed liquid cultures. Consistent with the growth reduction effect observed in the plate tests, *L. thermotolerans* grew much more efficiently than *Z. rouxii* in their mixed culture. Its success can be attributed to the fact that its cells depleted the medium of a substance that was needed by *Z. rouxii* for growth. A similar phenomenon may also account for the dominance of *Metschnikowia* over *Z. rouxii* (*Metschnikowia* depletes the medium of free ferric ions [96]), but interestingly, it did not result in significant growth arrest in the plate tests. Concerning the three major yeast species of the Essence wines, both tested *Candida* isolates overgrew *Z. rouxii* with a low sugar content but the opposite happened when the sugar content was high. The latter observation was consistent with the microplate results, which measured a higher growth rate for *Z. rouxii* at high sugar concentrations. However, it was less competitive than *Z. pseudobailii* and *H. osmophila* in the mixed culture at a high sugar content despite its higher osmotolerance in the microplate tests. When interpreting the experimental results, it should be borne in mind that the grape must is a much more complex environment than the laboratory media.

Interestingly, no *Saccharomyces* were found among the 3209 isolates, which is an unexpected finding because, in previous studies, we found *S. cerevisiae* and *S. uvarum* strains in botrytised grapes and high-sugar Tokaj wines [42,96,99] including Essence wines [49]. The absence of *Saccharomyces* may have been due to its lower osmotolerance but also to the common practice of treating the wines with sulphite against oxidation at times when the other local wines have completed fermentation but the Essence wines are still in an early stage of fermentation.

### 4.3. Intraspecies Clonal Diversity and Segregation

During the taxonomic identification of the isolates, we noticed that the number of phenotypic clusters was higher than the number of identified species. While isolates belonging to the same cluster were always conspecific, in certain cases, two or even more clusters turned out to belong to the same species. The colony morphology was one of the traits that showed intraspecies diversity. Previous studies reported on heterogeneous colony morphology in yeasts, but colony heterogeneity has not been reported yet for a population of the species found in the Essence wines. In *S. cerevisiae*, a complex genetic network determines the morphology of colonies, and changes in the network cause changes in the shape of the colonies [106]. Thus, the different morphologies of conspecific yeasts of the Essence wines are also likely to reflect genetic differences. Apart from morphological heterogeneity, the largest group of conspecific isolates, namely, the *Z. rouxii* strains, were also heterogeneous in their maltose utilisation, temperature sensitivity, tolerance to ethanol and sulphite (including in acid), H_2_S production and the growth rate at high sugar concentrations in the microplate tests. By definition, the species *Z. rouxii* is variable in the ability to grow at 37 °C and on maltose as a carbon source [107]. Consistent with this, we found both heat resistant and heat-sensitive strains, as well as maltose^+^ and maltose^−^ strains, among the isolates. Interestingly, there was no correlation between the two traits. No correlation was observed between the other variable strains either. The comparison of the karyotypes, microsatellite-primed RAPD (Random Amplification of Polymorphic DNA) patterns and the mtDNA RFLP (Restriction Fragment Length Polymorphism) patterns revealed an even higher heterogeneity in the genomes of the *Z. rouxii* isolates. Strains that were found to be similar in one test usually differed in other tests, and even strains isolated from the same wine turned out to be different when the results of all tests were taken into consideration. From the high heterogeneity of the isolates, we inferred that the *Z. rouxii* populations of the Essence wines consisted of subpopulations of different clones. However, when comparing the topologies of the dendrograms, one has to bear in mind that the positions of the strains do not necessarily reflect their real genetic relationships [108]. The populations of the other species also seemed to be heterogeneous because their strains were also diverse in terms of their colony morphology and certain physiological properties. The taxonomic identification of the isolates was based on D1/D2 sequencing because this domain is the most widely used barcode of the ascomycetous yeasts. With the exception of the *Metschnikowia* isolates and one *C. lactis-condensi* isolate, all conspecific isolates had identical D1/D2 sequences. However, they may not be uniform over the entire range of LSU (Large Subunit rRNA) genes. Sequencing of the rest of the genes could reveal variable positions and detect correlations between intraspecific morphological and physiological diversity and nucleotide substitutions in these positions.

The clonal heterogeneity could be attributed to multiple (recurrent) infections by heterogeneous populations of the species residing in the winery environment or coming with the harvested grapes. The alternative possibility is that the strains segregate during propagation in the wine. The morphologically different sectors in the *Z. rouxii*, *C. lactis-condensi*, *L. thermotolerans*, *Metschnikowia* and *P. membranifaciens* colonies and the segregation of the cultures of the *C. lactis-condensi* isolates into cells producing larger and smaller colonies on the nitrate medium (data not shown) corroborate this possibility. The evolution of strain diversity during vegetative propagation has been observed in both *S. cerevisiae* (e.g., [109,110,111,112]) and non-*Saccharomyces* species [113,114]. In *S. cerevisiae*, a process referred to as FAGE (fast adaptive genome evolution) was proposed to account for clonal changes in response to the drastically changing environment during fermentation [115]. However, it is not likely that a similar process takes place in the yeast biota of the Essence wines because the composition of these wines does not change significantly during fermentation. As far as we are aware, this is the first report on the clonal structures of *Z. rouxii*, *C. lactis-condensi* and *C. zemplinina* populations in fermenting wines.

## 5. Conclusions

1. The fermentation of high-sugar wine can take place in the absence of *Saccharomyces*.
2. Instead of *Saccharomyces*, osmotolerant “spoilage” yeasts can ferment when the sugar concentration is extremely high.
3. In botrytised Tokaj Essence wines of sugar concentrations ranging from 365 to 752 g∙L^−1^, *Zygosaccharomyces rouxii*, *Candida lactis-condensi* and *C. zemplinina* were the dominating species.
4. The minor species were either other “spoilage” yeasts or less osmotolerant biofilm-producing yeasts.
5. The high phenotypical and molecular (karyotype, mtDNA-RFLP and MSP-PCR) diversity of the conspecific strains indicates that diverse clones of the species coexisted in the wines.
6. Genetic segregation of certain clones and interaction of the species (antagonism and crossfeeding) could also shape the fermenting yeast biota.

## Figures and Tables

**Table 1:** List and properties of samples.

Sample	Location of Winery	Vintage	Stored in ^1^	Reducing Sugar g/L	Alcohol%	Extract		pH	Acid		SO_2_^2^		CFU ^3^ (10^8^/mL)
						Sugar-free (g/L)	Sum (g/L)		Titr. (g/L)	Vol. (g/L)	Free (mg/L)	All (mg/L)	
1	Vámosújfalu	1985	B	365.00	5.53	94.00	459.00	3.3	12.12	0.93	10	130	0
2	Tolcsva	2000	GB	711.60	1.12	99.60	811.20	3.26	13.96	0.39	8	24	46.5
3	Vámosújfalu	2005	B	498.10	3.64	60.90	559.00	3.12	12.12	0.90	10	248	0
4	Unspecified	2007	B	510.80	3.46	81.50	592.30	3.44	8.48	0.84	6	236	0
5	Hercegkút	2013	B	509.00	1.95	69.20	578.20	2.99	10.50	0.87	8	250	0.00045
6	Tokaj	2013	B	544.00	2.49	60.50	604.50	3.19	11.58	0.84	10	306	0
7	Unspecified	2017	GB	538.70	5.11	79.60	618.30	3.44	13.60	1.08	12	384	1
8	Unspecified	2017	OB	518.40	3.87	77.80	596.20	3.17	12.68	1.20	8	300	1
9	Mád	2017	B	620.10	2.76	55.60	675.70	3.32	12.45	0.05	6	18	6
10	Tolcsva	2017	B	426.90	4.76	43.80	470.70	3.39	9.96	0.69	6	16	0.0004
11	Tolcsva	2018	T	517.90	3.80	154.70	672.60	3.52	10.19	1.08	12	210	0.05
12	Mád	2018	B	752.20	2.60	41.80	794.00	3.24	11.68	0.54	10	20	1
13	Tolcsva	2019	GB	724.30	0.00	59.60	783.90	3.53	9.10	0.90	42	360	0
14	Tolcsva	2019	C	584.00	4.01	30.10	614.10	3.4	11.27	0.93	6	16	0.00035
15	Unspecified	2019	T	729.30	0.00	50.40	779.70	3.28	12.64	0.90	42	340	0.00005
16	Hercegkút	2019	T	620.10	1.68	46.00	666.10	3.71	9.56	0.78	6	30	102
17	Tolcsva	2019	GB	617.50	1.70	47.80	665.30	3.41	11.48	0.99	6	116	66
18	Tarcal	2017	GB	740.00	0.39	34.30	774.30	3.48	9.94	0.50	8	16	3
19	Tarcal	2019	GB	645.50	3.21	27.90	673.40	3.25	11.64	0.00	n.d.	n.d.	16
20	Mád	2019	GB	731.90	2.85	9.10	741.00	3.59	8.07	0.00	n.d.	n.d.	60
21	Bodrogolaszi	2013	B	533.70	3.24	90.30	624.00	3.42	9.37	0.35	n.d.	n.d.	0.00012
22	Bodrogolaszi	2016	GB	716.60	2.97	45.20	761.80	3.26	22.14	0.00	n.d.	n.d.	0.00052
23	Tolcsva	2016	T	493.00	4.15	141.40	634.40	3.3	19.98	0.53	n.d.	n.d.	0
24	Bodrogolaszi	2017	GB	655.60	3.01	33.40	689.00	3.74	10.92	0.05	n.d.	n.d.	40
25	Bodrogolaszi	2017	T	574.30	3.47	71.80	646.10	3.74	12.63	0.47	n.d.	n.d.	0.5
26	Mixed	2017	T	681.10	2.97	49.50	730.60	3.32	16.22	0.00	n.d.	n.d.	0.00025
27	Bodrogolaszi	2019	T	609.90	3.00	76.80	686.70	3.57	13.43	0.00	n.d.	n.d.	22.3
28	Mád	2013	GB	543.80	3.19	81.20	625.00	3.51	10.63	0.40	n.d.	n.d.	12

^1^ B: bottle, C: ceramic amphora, GB: glass balloon, OB: oak barrel, T: stainless steel tank; ^2^ n.d.: not determined; ^3^ CFU: colony-forming unit (viable cell).

**Table 2:** The minimal inhibitory concentrations (MICs) of glucose as determined in agar plate tests for representatives of each species.

Isolate	Species	MIC (% Glucose)
15-1	*H. osmophila*	>70
2-25	*K. fluxuum*	40
15-11, 16-33, G20-16	*L. thermotolerans*	>70
16-39, 20-1, G20-6	*M. pulcherrima* clade *sp.*	≤70
2-40, 2-65	*P. membranifaciens*	60
G10-1, 11-25, 12-63, 14-4, 20-18	*C. zemplinina*	>70
G8-1, 11-33	*C. lactis-condensi*	>70
G9-4,		60
9-1, 19-1		50
16-30	*Z. bailii*	>70
5-2, 5-43, G5-1	*Z. lentus*	>70
10-4	*Z. pseudobailii*	>70
All isolates	*Z. rouxii*	>70

**Table 3:** Phenotypic diversity of *Z. rouxii* isolates.

Isolate	Colony Morphology	Growth on/at ^1^						MIC			Acid Prod (mm)	BiGGY
		Mannitol	Galactose	Glycerol	Maltose	Lysine	37 °C	Glucose (%)	Ethanol (%)	Sulphur (mg∙L^−1^)		
9-3	Rough white	+	+	+	+	+	+	>70	14	300	3	3
9-78	Dull white	+	+	+	+	+	+	>70	14	300	3	3
9-85	Rough white	+	+	+	+	+	+	>70	14	300	3	3
G9-1	Dull white	+	+	+	+	+	+	>70	14	300	3.5	4
12-8	Dull white	+	+	+	+	+	+	>70	12	700	3	4
G12-1	Dull white	+	+	+	+	+	+	>70	12	700	3.5	4
20-2	Rough white	+	+	+	+	+	+	>70	14	700	2.5	4
12-1	Rough white	+	+	+	w	+	+	>70	14	400	3	3
15-2	Dull white	+	+	+	w	+	+	>70	12	800	2.5	3
15-5	Dull white	+	+	+	w	+	+	>70	14	800	2	3
G17-1	Dull white	+	+	+	w	+	+	>70	14	300	2	3
11-10	Dull white	+	+	+	−	+	+	>70	14	800	1.75	2
12-2	Dull white	+	+	+	−	+	+	>70	14	300	3	3
12-23	Dull white	+	+	+	−	+	+	>70	14	400	2	1
14-2	Dull white	+	+	+	−	+	+	>70	14	300	3	4
20-7	Rough white	+	+	+	−	+	+	>70	14	300	3	4
27-1	Dull white	+	+	+	+	+	w	>70	14	700	3	3
G16-1	Dull white	+	+	+	+	+	w	>70	12	700	2	4
G22-1	Dull white	+	+	+	+	+	−	>70	12	700	3	3
24-2	Dull white	+	+	+	+	+	−	>70	12	700	2.5	3
12-43	Rough white	+	+	+	+	w	−	>70	14	300	3	3
14-21c	White sectored	+	+	+	w	+	w	>70	12	700	2	4
17-14	Dull white	+	+	+	w	+	w	>70	12	800	1.5	3
G16-36	Rough white	+	+	+	w	+	−	>70	14	300	3	3
17-62	Dull white	+	+	+	w	+	−	>70	14	800	2	2
27-83	Rough white	+	+	+	w	+	−	>70	10	400	3.5	4
G21-21	Dull white	+	+	+	−	+	w	>70	14	700	3.5	3
2-1	Dull white	+	+	+	−	+	−	>70	14	700	3.5	3
2-3	Dull white	+	+	+	−	+	−	>70	14	700	3.5	3
2-42	Dull white	+	+	+	−	+	−	>70	14	600	3	3
G2-1	Dull white	+	+	+	−	+	−	>70	14	600	3	3
14-21a	Dull white	+	+	+	−	+	−	>70	13	300	3	4
14-53	Dull white	+	+	+	−	+	−	>70	12	300	3	4
G14-1	Dull white	+	+	+	−	+	−	>70	12	300	3	4
G20-1	Dull white	+	+	+	−	+	−	>70	12	400	3.5	4
G20-56	Rough white	+	+	+	−	+	−	>70	12	400	3.5	4
G24-10a	Dull white	+	+	+	−	+	−	>70	12	600	3	3
G27-1	Rough white	+	+	+	−	+	−	>70	14	700	3	3
12-30	Dull white	+	+	+	−	w	+	>70	14	400	2	1
19-6	Dull white	+	+	+	−	w	w	>70	12	800	3	1
12-18	Dull white	+	+	+	−	w	−	>70	14	400	3	1
G19-1	Dull white	+	+	+	−	w	−	>70	14	800	2	1
28-1	Dull white	w	w	w	w	w	−	>70	12	400	2	1
28-5	Dull white	w	w	w	w	w	−	>70	12	400	2.5	1
28-58	Dull white	w	w	w	w	w	−	>70	12	600	1	1
G28-1	Dull white	w	w	w	w	w	−	>70	12	600	1	1

^1^+: growth, −: no growth, w: weak growth.

**Table 4:** Interactions of isolates on agar plates. Results of two experiments.

Colony		Width of Inhibition Zones (mm) in the Lawn of																
		G8-1	G11-4	11-25	17-1	15-1	2-25	15-11	16-33	20-1	16-39	2-65	2-40	2-3	20-2	16-30	5-2	10-4
Isolate	Species	*C. lactis-condensi*	*C. lactis-condensi*	*C. zemplinina*	*C. zemplinina*	*H. osmophila*	*K. fluxuum*	*L. thermotolerans*	*L. thermotolerans*	*M. p.* clade *sp.*	*M. p.* clade *sp.*	*P. membranifaciens*	*P. membranifaciens*	*Z. rouxii*	*Z. rouxii*	*Z. bailii*	*Z. lentus*	*Z. pseudobailii*
G8-1	*C. lactis-condensi*	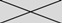	−	−	−	−	−	−	−	−	−	−	−	−	−	−	−	−
G11-4	*C. lactis-condensi*	−	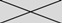	−	−	−	−	−	−	−	−	−	−	−	−	−	−	−
11-25	*C. zemplinina*	−	2, 2	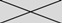	−	3, 3	−	2,2	−	−	−	−	−	−	−	−	−	−
17-1	*C. zemplinina*	−	−	−	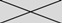	3, 2	crossf	2,2	−	−	−	−	−	−	−	−	−	−
15-1	*H. osmophila*	−	−	−	−	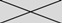	−	−	−	−	−	−	−	−	−	−	−	−
2-25	*K. fluxuum*	−	−	−	−	−	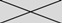	crossf	crossf	−	−	−	−	−	−	−	−	−
15-11	*L. thermotolerans*	2, 1	3, 3	2, 2	1, 1	2, 1	2, 2	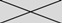	2, 0	2, 2	2, 2	2, 1	1, 1	1, 0	2, 2	2, 2	2, 2	1, 1
16-33	*L. thermotolerans*	1, 1	2, 2	2, 2	2, 1	1, 1	2, 2	2, 2	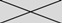	3, 0	2, 2	−	−	1, 1	2, 2	2, 1	1, 1	1, 1
20-1	*M. pulcherrima* clade *sp.*	1, 0	1, 1	−	1, 0	−	crossf	crossf	−	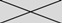	−	−	−	−	1, 1	−	−	−
16-39	*M. pulcherrima* clade *sp.*	−	1, 1	−	1, 1	−	−	−	crossf	−	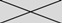	0.5, 1	0.5, 1	−	1, 0	−	−	−
2-65	*P. membranifaciens*	−	1, 0	−	−	−	−	−	crossf	−	−	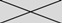	−	−	−	−	−	−
2-3	*Z. rouxii*	−	1, 0	−	−	1, 0	1, 0	−	−	−	−	1, 1	1, 0.5	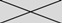	−	−	−	−
20-2	*Z. rouxii*	−	3, 3	−	−	−	1, 0	crossf	crossf	−	−	1, 1	1, 1	−	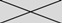	−	−	−
16-30	*Z. bailii*	−	1, 0	−	−	−	−	crossf	crossf	−	−	0.5, 0	−	−	−	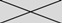	−	−
5-2	*Z. lentus*	−	1, 0	−	−	−	−	crossf	crossf	−	−	1, 0	0.5, 0	−	−	−	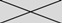	−
10-4	*Z. pseudobailii*	−	−	−	−	−	−	crossf	crossf	−	−	−	−	−	−	−	−	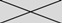

Crossf: crossfeeding.

**Table 5:** Growth in mixed cultures.

Mixed Cultures		Proportion of Isolates after 48 h of Incubation at	
Isolates	Species Combination	2% Glucose	30% Glucose
2-3 + 10-4	*Z. rouxii* + *Z. pseudobailii*	1:1.03	1:8
2-3 + 16-30	*Z. rouxii* + *Z. bailii*	n.d.	1.18:1
2-3 + G11-4	*Z. rouxii* + *C. lactis-condensi*	1:1.19	15.25:1
2-3 + 11-25	*Z. rouxii* + *C. zemplinina*	1:24	2.4:1
11-25 + G11-4	*C. zemplinina* + *C. lactis-condensi*	1:1.13	1:5.4
2-3 + 15-1	*Z. rouxii* + *H osmophila*	1:75	1:14.3
2-3 + 15-11	*Z. rouxii* + *L. thermotolerans*	1:>57	n.d
2-3 + 16-39	*Z. rouxii* + *Metschnikowia*	1:>84	n.d.
2-3 + 2-65	*Z. rouxii* + *P. membranifaciens*	1:>28	n.d.

n.d.: not determined.

## Data Availability

The data presented in this study are available in Csoma, H.; Kallai, Z.; Antunovics, Z.; Centye, K.; Sipiczki, M.; Vinification without Saccharomyces: interacting osmotolerant and “spoilage” yeast communities in fer-menting and ageing botrytized high-sugar wines (Tokaj Essence); Microorganisms and its supplementary ma-terial.

## Supplementary Materials

The following are available online at https://www.mdpi.com/2076-2607/9/1/19/s1: Figure S1. Growth of isolates in artificial must at high concentrations of sugar, Figure S2. UPGMA (Unweighted pair Group Method with Arithmetic means) dendrogram of mtDNA restriction fragment length polymorphism (RFLP) patterns of the *Z. rouxii* isolates, Figure S3. UPGMA dendrogram of the karyotypes of the *Z. rouxii* isolates, Table S1. List of taxonomically identified isolates, Table S2. Growth of isolates in artificial must in microplates at high concentrations of sugar.
