# Supplementary material for: Vinification without Saccharomyces: Interacting Osmotolerant and “Spoilage” Yeast Communities in Fermenting and Ageing Botrytised High-Sugar Wines (Tokaj Essence)
Source: Microorganisms. 2020 Dec 23;9(1):19. doi: 10.3390/microorganisms9010019 (PMC7822429; doi:10.3390/microorganisms9010019)
Supplement: Supplementary file 1 [file microorganisms-09-00019-s001.zip › microorganisms-1038291-supplementary/Fig. S1.pdf]

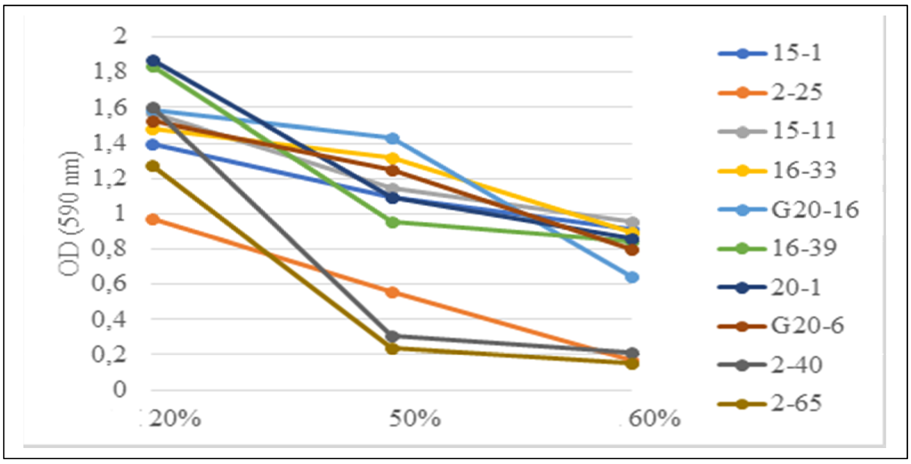

15-1 *H. osmophila*
2-25 *K. fluxuum*
15-11 *L. thermotholerans*
16-33 *L. therotholerans*
g20-16 *L. thermotholerans*
16-39 *Metschnikowia*
20-1 *Metschnikowia*
g20-6 *Metschnikowia*
2-40 *P. membranifaciens*
2-65 *P. membranifaciens*

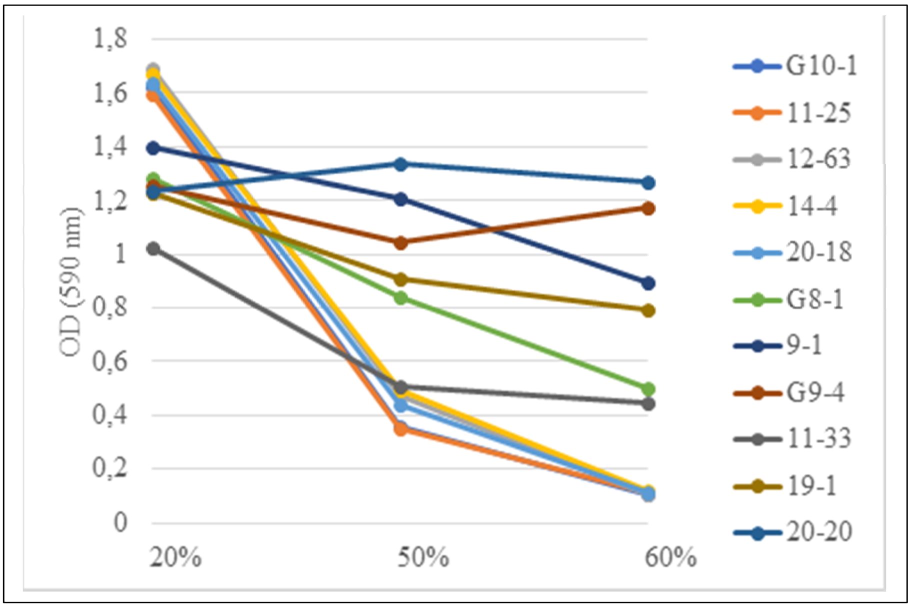

g10-1 *C. zemplinina*
11-25 *C. zemplinina*
12-63 *C. zemplinina*
14-4 *C. zemplinina*
20-18 *C. zemplinina*
g8-1 *C. lactis-condensi*
9-1 *C. lactis-condensi*
g9-4 *C. lactis-condensi*
11-33 *C. lactis-condensi*
19-1 *C. lactis-condensi*
20-20 *C. apicola*

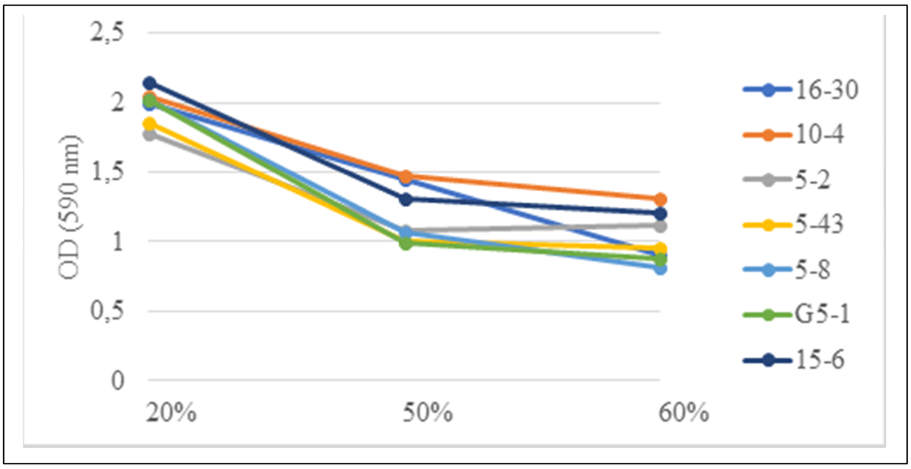

16-30 *Z. bailii*
10-4 *Z. pseudobailii*
5-2 *Z. lentus*
5-43 *Z. lentus*
5-8 *Z. lentus*
g5-1 *Z. lentus*
15-6 *Z. bisporus*

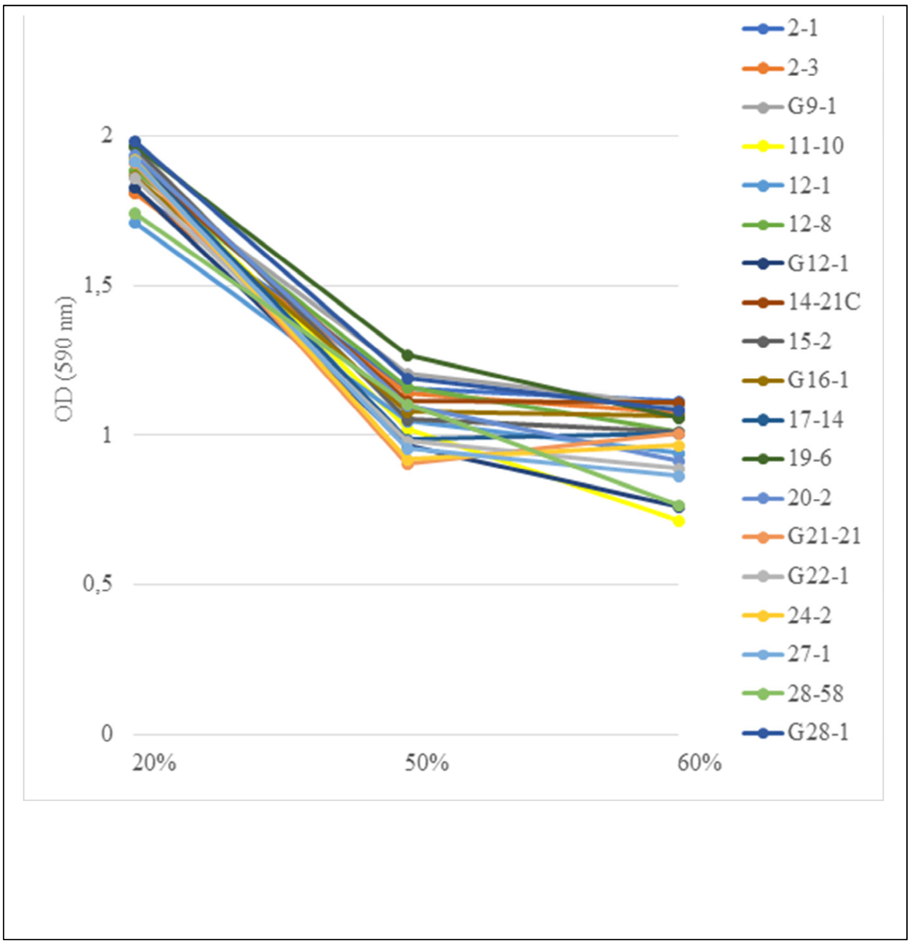

All *Z. rouxii*

Figure S1. Growth of isolates in artificial must at high concentrations of sugar.
