# Supplementary material for: Vinification without Saccharomyces: Interacting Osmotolerant and “Spoilage” Yeast Communities in Fermenting and Ageing Botrytised High-Sugar Wines (Tokaj Essence)
Source: Microorganisms. 2020 Dec 23;9(1):19. doi: 10.3390/microorganisms9010019 (PMC7822429; doi:10.3390/microorganisms9010019)
Supplement: Supplementary file 1 [file microorganisms-09-00019-s001.zip › microorganisms-1038291-supplementary/Fig. S3.pdf]

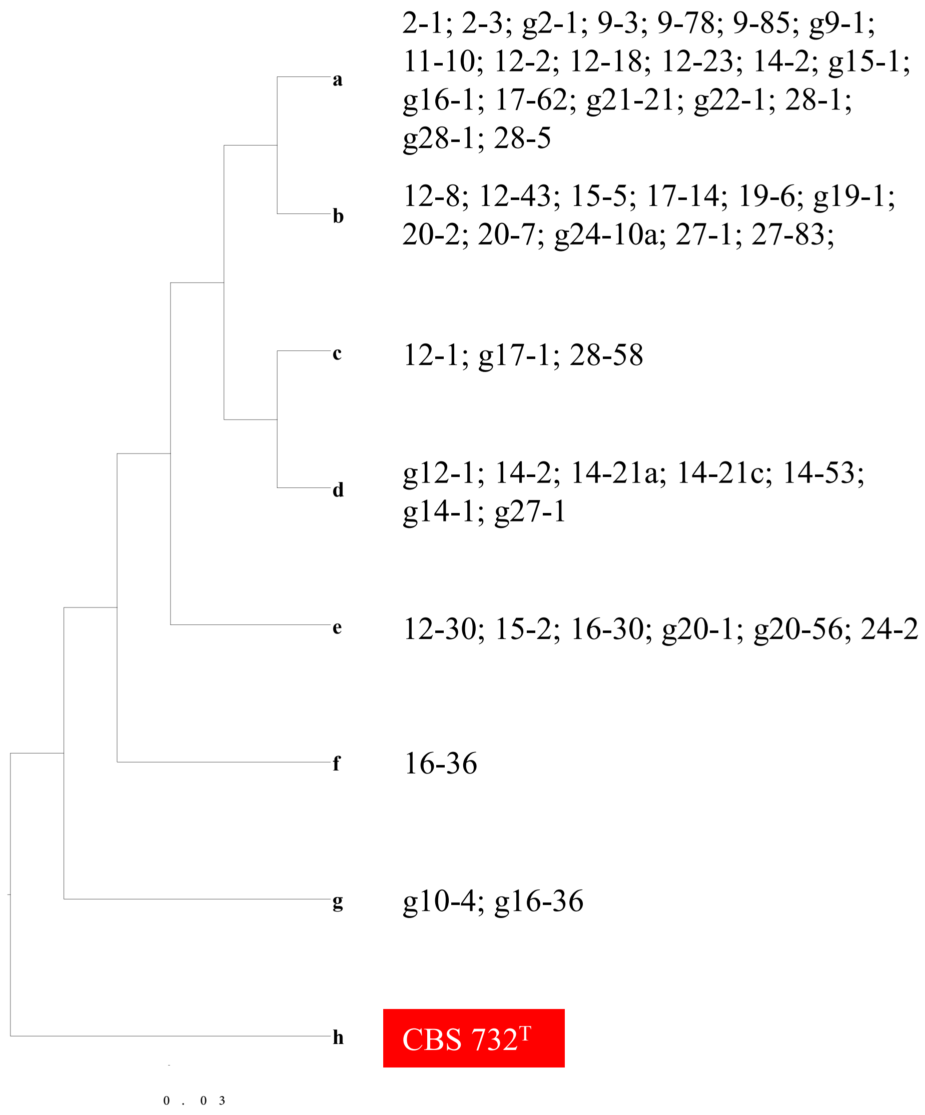

Figure S3. UPGMA (Unweighted Pair Group Method with Arithmetic means) dendrogramme of the karyotypes of the *Z. rouxii* isolats
