# Supplementary material for: Vinification without Saccharomyces: Interacting Osmotolerant and “Spoilage” Yeast Communities in Fermenting and Ageing Botrytised High-Sugar Wines (Tokaj Essence)
Source: Microorganisms. 2020 Dec 23;9(1):19. doi: 10.3390/microorganisms9010019 (PMC7822429; doi:10.3390/microorganisms9010019)
Supplement: Supplementary file 1 [file microorganisms-09-00019-s001.zip › microorganisms-1038291-supplementary/Table S1.pdf]

Table S1. List of taxonomically identified isolates.

Isolate	Growth morphology on YEA2	Utilisation of												Growth at 37 °C	Taxonomic affiliation based on D1/D2 sequence identity with type strains	
		Saccharose	Raffinose	Arabinose	Cellobiose	Mannitol	Galactose	Glycerol	Maltose	Vitamin-	Nitrate	Lysine	Acetic acid		Most similar species	D1/D2 identity % (with type strain)
2-1	Dull white	w	-	-	-	+	+	+	-	w	w	+	-	-	*Z. rouxii*	100
2-3	Dull white	w	-	-	-	+	+	+	-	w	w	+	-	-	*Z. rouxii*	100
2-25	Dull brownish, lobed	-	-	-	-	+	-	w	w	+	-	+	+	-	*K. fluxuum*	100
2-40	Dull brownish	-	-	-	-	-	-	+	w	+	-	+	-	-	*P. membranifaciens*	100
2-42	Dull white	w	-	-	-	+	+	+	-	w	w	+	-	-	*Z. rouxii*	100
2-65	Dull brownish	+	+	-	-	-	+	+	-	+	w	+	-	-	*P. membranifaciens*	100
G2-1	Dull white	w	-	-	-	+	+	+	-	w	w	+	-	-	*Z. rouxii*	100
G2-58	Dull white		-	-	-	+	+	+	-	w	w	+		-	*Z. rouxii*	100
5-2	Dull white	+	-	-	-	-	-	w	-	w	-	-	+	-	*Z. lentus*	100
5-8	Dull white	w	-	-	-	+	+	+	-	w	w	-	+	+	*Z. lentus*	100
5-43	Dull white	+	-	-	-	-	-	-	-	w	-	-	+	-	*Z. lentus*	100
G5-1	Dull white	+	+	+	+	+	+	+	+	+	w	+	+	-	*Z. lentus*	100
7-1	Thin butyrous	w	w	-	-	-	-	-	-	-	w	w	-	-	*C. lactis-condensi*	100
7-10	Thin butyrous lobed	w	w	-	-	-	-	-	-	-	w	w	-	-	*C. lactis-condensi*	100
G7-1	Thin butyrous	w	w	-	-	-	-	-	-	-	w	w	-	-	*C. lactis-condensi*	100
8-1	Thin butyrous	w	w	-	-	-	-	-	-	-	w	w	-	-	*C. lactis-condensi*	99.79
G8-1	Thin, sectored	w	w	-	-	-	-	-	-	-	w	w	-	-	*C. lactis-condensi*	100
9-1	Thin butyrous	w	w	-	-	-	-	-	-	-	w	+	-	-	*C. lactis-condensi*	100
9-3	Rough white	w	-	-	-	+	+	+	+	w	w	+	-	-	*Z. rouxii*	100
9-78	Dull white	w	-	-	-	+	+	+	+	w	w	+	-	+	*Z. rouxii*	100
9-85	Rough white	w	-	-	-	+	+	+	+	w	w	+	-	+	*Z. rouxii*	100
G9-1	Dull white	w	-	-	-	+	+	+	+	w	w	+	-	+	*Z. rouxii*	100
G9-4	Thin butyrous	w	w	-	-	-	-	-	-	-	+	+	-	-	*C. lactis-condensi*	100
10-1	Thin glistering	+	+	-	-	-	-	-	-	-	-	w	-	-	*C. zemplinina*	100
10-4	Dull white	+	+	-	w	+	w	+	w	+	-	-	-	w	*Z. pseudobailii*	100
G10-1	Thin	+	w	-	-	-	-	-	-	-	w	-	-	-	*C. zemplinina*	100
11-1	Thin butyrous	w	w	-	-	-	-	-	-	-	+	+	-	-	*C. lactis-condensi*	100
11-10	Dull white	w	-	-	-	+	+	+	-	w	w	+	-	+	*Z. rouxii*	100

11-25	Thin glistering	+	+	-	-	-	-	-	-	-	w	+	-	-	*C. zemplinina*	100
11-33	Thin butyrous	w	-	-	-	-	-	-	-	-	w	w	-	-	*C. lactis-condensi*	100
11-76	Thin sectored	w	w	-	-	-	-	-	-	-	w	w	-	-	*C. lactis-condensi*	100
G11-4	Thin sectored	w	-	-	-	-	-	-	-	-	+	w	-	-	*C. lactis-condensi*	100
12-1	Rough white	w	-	-	-	+	+	+	w	w	w	+	-	+	*Z. rouxii*	100
12-2	Dull white	w	-	-	-	+	+	+	-	w	w	+	-	+	*Z. rouxii*	100
12-8	Dull white	w	-	-	-	+	+	+	+	w	w	+	-	+	*Z. rouxii*	100
12-18	Dull white	w	-	-	-	+	+	+	-	w	w	w	-	-	*Z. rouxii*	100
12-23	Dull white	w	-	-	-	+	+	+	-	w	w	+	-	+	*Z. rouxii*	100
12-30	Dull white	w	-	-	-	+	+	+	-	w	w	w	-	+	*Z. rouxii*	100
12-43	Rough white	w	-	-	-	+	+	+	+	w	w	w	-	-	*Z. rouxii*	100
12-63	Thin listering lobed	+	w	-	-	-	-	-	-	-	w	+	-	-	*C. zemplinina*	100
G12-1	Dull white	w	-	-	-	+	+	+	+	w	w	+	-	+	*Z. rouxii*	100
14-1	Thin glistering	+	+	-	-	-	-	-	-	-	-	+	-	-	*C. zemplinina*	100
14-2	Dull white	w	-	-	-	+	+	+	-	w	w	+	-	+	*Z. rouxii*	100
14-4	Thin glistering	+	w	-	-	-	-	-	-	-	w	+	+	-	*C. zemplinina*	100
14-21a	Dull white	w	-	-	-	+	+	+	-	w	w	+	-	-	*Z. rouxii*	100
14-21c	White sectored	w	-	-	-	+	+	+	w	w	w	+	-	w	*Z. rouxii*	100
14-53	Dull white	w	-	-	-	+	+	+	-	w	w	+	-	-	*Z. rouxii*	100
G14-1	Dull white	w	-	-	-	+	+	+	-	w	w	+	-	-	*Z. rouxii*	100
G14-16	Thin glistering sectored	w	w	-	-	-	-	-	-	-	w	-	-	-	*C. zemplinina*	100
15-1	Cream coloured	+	w	-	+	-	-	-	w	w	-	+	-	-	*H. osmophila*	100
15-2	Dull white	w	-	-	-	+	+	+	w	w	w	+	-	+	*Z. rouxii*	100
15-5	Dull white	w	-	-	-	+	+	+	w	w	w	+	-	-	*Z. rouxii*	100
15-6	Dull white	+	w	-	-	+	w	+	w	+	-	+	+	-	*Z. bisporus*	100
15-11	Cream coloured glossy	+	w	-	-	+	+	+	+	+	-	+	-	w	*L. thermotholerans*	100
16-1	Thin glistering	+	+	-	-	-	-	-	-	-	w	+	d-	-	*C. zemplinina*	100
16-2	Thin glistering	+	+	-	-	-	-	-	-	-	w	+	d-	-	*C. zemplinina*	100
16-30	Dull white	+	+	-	-	+	+	w	-	w	w	w	+	+	*Z. baili*	100
16-33	Brownish glossy	+	+	-	-	+	+	+	+	+	w	+	-	+	*L. thermotolerans*	100
16-39	Maroon	+	+	-	+	+	+	+	+	+	w	+	-	+	*M. pulcherrima* clade *sp.*	97-99
16-43	Dull white	+	+	-	-	+	+	w-	-	+	w	-	+	+	*Z. bailii*	100
16-51	Thin white	+	+	-	-	-	-	-	-	-	-	-	-	-	*C. zemplinina*	100
G16-1	Dull white	w	-	-	-	+	+	+	+	w	w	+	-	w	*Z. rouxii*	100
G16-36	Rough white	w	-	-	-	+	+	+	w	w	w	+	-	-	*Z. rouxii*	100
G16-37	Thin glistering	+	+	-	-	-	-	-	-	-	-	w	-	-	*C. zemplinina*	100
17-1	Thin glistering	+	w	-	-	-	-	-	-	-	-	-	-	-	*C. zemplinina*	100

17-14	Dull white	w	-	-	-	+	+	+	w	w	w	+	-	w	*Z. rouxii*	100
17-35	Thin glistering	+	+	-	-	-	-	-	-	-	w	w	-	-	*C. zemplinina*	100
17-62	Dull white	w	-	-	-	+	+	+	w	w	w	+	-	-	*Z. rouxii*	100
G17-1	Dull white	w	-	-	-	+	+	+	w	w	w	+	-	+	*Z. rouxii*	100
18-42	Thin glistering	+	w	-	-	-	-	-	-	-	+	-	-	-	*C. zemplinina*	100
G17-2	Thin glistering	+	w	-	-	-	-	-	-	-	+	-	-	-	*C. zemplinina*	100
18-2	Thin butyrous	w	w	-	-	-	-	-	-	-	w	+	-	-	*C. lactis-condensi*	100
18-5	Thin butyrous	w	w	-	-	-	-	-	-	-	w	+	-	-	*C. lactis-condensi*	100
G18-1	Thin butyrous	w	w	-	-	-	-	-	-	-	+	+	-	-	*C. lactis-condensi*	100
19-1	Thin butyrous lobed	+	w	-	-	-	-	-	-	-	+	w	-	-	*C. lactis-condensi*	100
19-6	Dull white	w	-	-	-	+	+	+	-	w	w	w	-	-	*Z. rouxii*	100
19-13	Thin glistering	+	w	-	-	-	-	-	-	-	+	-	-	-	*C. zemplinina*	100
19-62	Thin glistering	+	+	-	-	-	-	-	-	-	-	w	-	-	*C. zemplinina*	100
19-65	Coral glistering	+	+	-	w	w	+	-	+	-	-	-	-	-	*R. mucilaginosa*	100
19-78	Dull brownish	w	-	-	-	+	+	+	-	w	w	+	-	-	*Z. rouxii*	100
G19-1	Dull white	w	-	-	-	+	+	+	-	w	w	w	-	-	*Z. rouxii*	100
G19-6	Thin glistering	+	+	-	-	-	-	-	-	-	+	+	-	-	*C. lactis-condensi*	100
20-1	Maroon	-	w	-	+	+	+	+	+	+	w	+	-	+	*M. pulcherrima* clade *sp.*	97-99
20-2	Rough white	w	-	-	-	+	+	+	+	w	w	+	-	+	*Z. rouxii*	100
20-7	Rough white	w	-	-	-	+	+	+	-	w	w	+	-	+	*Z. rouxii*	100
20-14	White sectored	w	-	-	-	w	+	+	-	+	w	-	-	+	*Z. rouxii*	100
20-18	Thin glistering	+	W	-	-	-	-	-	-	-	-	-	-	-	*C. zemplinina*	100
20-20	Faintly yellow	w	w	-	-	+	-	+	-	+	w	-	-	-	*C. apicola*	100
G20-1	Dull white	w	-	-	-	+	+	+	-	w	w	+	-	-	*Z. rouxii*	100
G20-4	Thin butyrous	w	w	-	-	-	-	-	-	-	w	w	-	-	*C. lactis-condensi*	100
G20-6	Maroon	+	w	-	+	+	+	+	+	+	+	+	-	+	*M. pulcherrima* clade *sp.*	97-99
G20-16	Yellowish glossy	+	w	-	-	w	+	w	+	w	+	+	-	+	*L. thermotolerance*	100
G20-41	Maroon	+	w	-	+	+	+	+	+	+	+	+	-	+	*M. pulcherrima* clade *sp.*	97-99
G20-56	Rough white		-	-	-	+	+	+	-	w	w	+	-	-	*Z. rouxii*	100
21-1	Thin butyrous lobed	w	w	-	-	-	-	-	-	-	w	w	-	-	*C. lactis-condensi*	100
21-25	Thin butyrous lobed	w	w	-	-	-	-	-	-	-	w	w	-	-	*C. lactis-condensi*	100
G21-1	Thin butyrous	w	w	-	-	-	-	-	-	-	w	w	-	-	*C. lactis-condensi*	100
G21-7	Thin butyrous	w	w	-	-	-	-	-	-	-	w	w	-	-	*C. lactis-condensi*	100
G21-21	Dull white	w	-	-	-	+	+	+	-	w	w	+	-	w	*Z. rouxii*	100
22-6	Dull white	w	-	-	-	+	+	+	w	w	w	+	-	-	*Z. rouxii*	100
22-26	Dull white	w	-	-	-	+	+	+	+	w	w	+	-	-	*Z. rouxii*	100
G22-1	Dull white	w	-	-	-	+	+	+	+	w	w	+	-	-	*Z. rouxii*	100

24-1	Thin butyrous lobed	w	w	-	-	-	-	-	-	-	+	w	-	-	*C. lactis-condensi*	100
24-2	Dull white	w	-	-	-	+	+	+	+	w	w	+	-	-	*Z. rouxii*	100
G24-1	Thin butyrous	w	w	-	-	-	-	-	-	-	w	w	-	-	*C. lactis-condensi*	100
G24-10a	Dull white	w	-	-	-	+	+	+	-	w	w	+	-	-	*Z. rouxii*	100
G24-10d	Thin butyrous	w	w	-	-	-	-	-	-	-	w	w	-	-	*C. lactis-condensi*	100
25-1	Thin butyrous	w	w	-	-	-	-	-	-	-	w	w	-	-	*C. lactis-condensi*	100
G25-1	Thin butyrous, lobed	w	w	-	-	-	-	-	-	-	w	w	-	-	*C. lactis-condensi*	100
26-2	Thin butyrous	w	w	-	-	-	-	-	-	-	+	w	-	-	*C. lactis-condensi*	100
G26-3	Glistering	w	w	-	-	-	-	-	-	-	+	w	-	-	*C. lactis-condensi*	100
27-1	Dull white	w	-	-	-	+	+	+	-	w	w	+	-	-	*Z. rouxii*	100
27-39	Thin butyrous,	w	w	-	-	-	-	-	-	-	-	w	w	-	*C. lactis-condensi*	100
27-83	Rough white	w	-	-	-	+	+	+	w	w	w	+	-	-	*Z. rouxii*	100
G27-1	Rough white	w	-	-	-	+	+	+	-	w	w	+	-	w	*Z. rouxii*	100
28-1	Dull white	w	-	-	-	w	w	w	w	w	w	w	-	-	*Z. rouxii*	100
28-58	Dull white	w	-	-	-	w	w	w	w	w	w	w	-	-	*Z. rouxii*	100
28-5	Dull white	w	-	-	-	w	w	w	w	w	w	w	-	-	*Z. rouxii*	100
G28-1	Dull white	w	-	-	-	w	w	w	w	w	w	w	-	-	*Z. rouxii*	100

+: growth; w: weak growth; -: no growth

C: *Candida; H*: Hanseniaspora*; K*: *Kregervanrija; L*: *Lachancea; M*: *Metschnikowia; R: Rhodotorula; P*: *Pichia;*
Z: *Zygosaccharomyces*
