# Supplementary material for: Vinification without Saccharomyces: Interacting Osmotolerant and “Spoilage” Yeast Communities in Fermenting and Ageing Botrytised High-Sugar Wines (Tokaj Essence)
Source: Microorganisms. 2020 Dec 23;9(1):19. doi: 10.3390/microorganisms9010019 (PMC7822429; doi:10.3390/microorganisms9010019)
Supplement: Supplementary file 1 [file microorganisms-09-00019-s001.zip › microorganisms-1038291-supplementary/Table S2.pdf]

Table S1. Growth of isolates in artificial must in microplates at high concentrations of sugar

Isolate	Species	Density of the culture after 119 h 27 min.					
		20% sugar		50% sugar		60% sugar	
		OD	St. dev	OD	St. dev.	OD	St. dev.
20-20	*C.. apicola*	1.236	0.041	1.341	0.149	1.266	0.050
G10-1	*C. zemplinina*	1.622	0.090	0.360	0.011	0.108	0.001
11-25	*C. zemplinina*	1.596	0.029	0.350	0.014	0.113	0.001
12-63	*C. zemplinina*	1.690	0.036	0.473	0.009	0.106	0.002
14-4	*C. zemplinina*	1.668	0.048	0.491	0.059	0.117	0.006
20-18	*C. zemplinina*	1.640	0.038	0.443	0.052	0.114	0.001
G8-1	*C. lactis-condensi*	1.283	0.100	0.842	0.065	0.504	0.043
9-1	*C. lactis-condensi*	1.396	0.098	1.210	0.009	0.895	0.106
G9-4	*C. lactis-condensi*	1.254	0.031	1.044	0.054	1.175	0.071
11-33	*C. lactis-condensi*	1.028	0.146	0.505	0.072	0.448	0.080
19-1	*C. lactis-condensi*	1.231	0.007	0.911	0.054	0.795	0.085
15-1	*H. osmophila*	1.393	0.048	1.093	0.158	0.910	0.025
2-25	*K. fluxuum*	0.974	0.143	0.554	0.049	0.169	0.003
15-11	*L. thermotholerans*	1.572	0.095	1.142	0.013	0.956	0.025
16-33	*L. therotholerans*	1.484	0.020	1.318	0.025	0.893	0.041
G20-16	*L. thermotholerans*	1.584	0.032	1.428	0.075	0.647	0.001
16-39	*M. pulcherrima* clade *sp.*	1.833	0.043	0.956	0.103	0.845	0.197
20-1	*M. pulcherrima* clade *sp.*	1.869	0.046	1.098	0.012	0.861	0.003
G20-6	*M. pulcherrima* clade *sp.*	1.528	0,018	1.253	0.210	0.800	0.023
2-40	*P. membranifaciens*	1.602	0.005	0.304	0.136	0.216	0.005
2-65	*P. membranifaciens*	1.278	0.024	0.236	0.004	0.151	0.001
16-30	*Z. bailii*	1.999	0.005	1.449	0.170	0.903	0.005
15-6	*Z. bisporus*	2.142	0.134	1.315	0.047	1.211	0.009
5-2	*Z. lentus*	1.775	0.031	1.078	0.119	1.121	0.125
5-43	*Z. lentus*	1.861	0.023	1.012	0.001	0.957	0.023
5-8	*Z. lentus*	2.014	0.038	1.070	0.084	0.814	0.015
G5-1	*Z. lentus*	2.015	0.058	0.991	0.014	0.883	0.055
10-4	*Z. pseudobailii*	2.041	0.206	1.469	0.179	1.313	0.044
2-1	*Z. rouxii*	1.816	0.034	1.154	0.086	1.115	0.051
2-3	*Z. rouxii*	1.808	0.028	1.138	0.035	1.073	0.011
G9-1	*Z. rouxii*	1.883	0.014	1.205	0.062	1.101	0.018
11-10	*Z. rouxii*	1.920	0.018	1.021	0.106	0.717	0.013
12-1	*Z. rouxii*	1.711	0.038	1.049	0.006	0.941	0.018
12-8	*Z. rouxii*	1.885	0.036	1.161	0.020	1.014	0.041
G12-1	*Z. rouxii*	1.832	0.029	0.965	0.111	0.763	0.105
14-21C	*Z. rouxii*	1.917	0.007	1.117	0.106	1.109	0.057
15-2	*Z. rouxii*	1.965	0.097	1.052	0.085	1.013	0.040
G16-1	*Z. rouxii*	1.864	0.106	1.08	0.062	1.061	0.039
17-14	*Z. rouxii*	1.929	0.012	0.985	0.051	1.005	0.011
19-6	*Z. rouxii*	1.967	0.021	1.268	0.033	1.058	0.006
20-2	*Z. rouxii*	1.939	0.041	1.100	0.185	0.915	0.038
G21-21	*Z. rouxii*	1.910	0.028	0.905	0.063	1.007	0.052
G22-1	*Z. rouxii*	1.859	0.043	0.980	0.052	0.891	0.033
24-2	*Z. rouxii*	1.925	0.005	0.922	0.068	0.965	0.002
27-1	*Z. rouxii*	1.915	0.025	0.957	0.059	0.863	0.023
28-58	*Z. rouxii*	1.744	0.033	1.105	0.050	0.768	0.032
G28-1	*Z. rouxii*	1.985	0.003	1.190	0.027	1.084	0.009
